# Local Pair Natural Orbital-Based Coupled-Cluster Theory through Full Quadruples (DLPNO–CCSDTQ)

**DOI:** 10.1021/acs.jctc.5c01910

**Published:** 2026-03-06

**Authors:** Andy Jiang, Devin A. Matthews, David Poole, Connor G. Briggs, Justin M. Turney, C. David Sherrill, Henry F. Schaefer III

**Affiliations:** † Center for Computational Quantum Chemistry, Department of Chemistry, 1355 University of Georgia, Athens, Georgia 30602, United States of America; ‡ Department of Chemistry, 171725 Southern Methodist University, Dallas, Texas 75275, United States of America; § Center for Computational Molecular Science and Technology, School of Chemistry and Biochemistry, School of Computational Science and Engineering, 1372 Georgia Institute of Technology, Atlanta, Georgia 30332-0400, United States

## Abstract

In this work, we implement a local pair natural orbital-based coupled-cluster method through the full treatment of quadruple excitations (CCSDTQ). The domain-based local pair natural orbital (DLPNO) approach, which has successfully been applied to lower levels of coupled-cluster theory, is utilized in our algorithm, and thus our algorithm is called DLPNO-CCSDTQ. For simplicity in the working equations and in the implementation, we *t*_1_-dress the two-electron integrals as well as Fock matrix elements. Our method can recover CCSDTQ-CCSDT and CCSDTQ-CCSDT­(Q) energy differences on the order of 0.01–0.05 kcal mol^–1^, even at a loose quadruples natural orbital (QNO) occupation number cutoff of 3.33 × 10^–6^. To highlight the capabilities of our code and its potential future applications, we showcase computations that would be intractable with canonical CCSDTQ, such as the benzene dimer, (H_2_O)_17_, and adamantane. With sufficient computing resources, computations up to 15 heavy atoms (40 atoms overall) may be feasible for fully bonded 3D systems.

## Graphical abstract

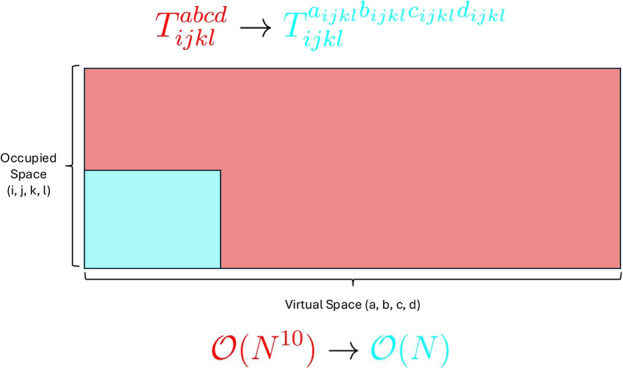

## 1 Introduction

Coupled-cluster (CC) theory^1,2^ has been one of the most powerful and reliable approaches utilized in *ab initio* electronic structure theory, allowing for a size-extensive approximation of the full configuration interaction (FCI)^3,4^ energy at any level of truncation. FCI represents the exact solution to the nonrelativistic time-independent Schrödinger equation for a given finite basis set within the Born–Oppenheimer approximation. The formalism for coupled-cluster theory is based on the exponential ansatz:

1 $$\left|\Psi_{\mathrm{CC}}\right\rangle =e^{T}\left|\Psi_{0}\right\rangle$$

2 $$E_{\mathrm{CC}}=\left\langle \Psi_{0}\left|e^{-T}Ĥe^{T}\right|\Psi_{0}\right\rangle$$

In these equations, |Ψ_0_⟩ represents a single-reference Hartree–Fock (HF) determinant. The cluster operator *T* can be expressed as a sum of operators *T*_*n*_ that generates *n*th-order excited determinants. The operator can be truncated in any desired order. This truncation creates a hierarchy of coupled-cluster methods that approach the FCI limit, typically with rapid convergence.^5–10^ The CCSDTQ method, or coupled-cluster with single, double, triple, and quadruple excitations,^11–14^ includes all electronic excitations from the reference determinant up to four electrons.

3 $$\left|\Psi_{\mathrm{CCSDTQ}}\right\rangle =e^{T_{1}+T_{2}+T_{3}+T_{4}}\left|\Psi_{0}\right\rangle$$

The exponential ansatz gives coupled-cluster methods size-extensivity, as higher order excitations are not completely neglected but expressed as products of lower-level cluster operators. For example, CCSDTQ approximates hextuple excitations through terms such as *T̂*_4_ *T̂*_2_, ${\mathrm{T̂}}_{3}^{2}$ , ${\mathrm{T̂}}_{2}^{3}$ , etc., which arise from a Taylor series expansion of $e^{T_{1}+T_{2}+T_{3}+T_{4}}$ . These are known as “disconnected” excitations. For a given level of truncation *n*, all excitations up to the order *n* are treated as a combination of “connected” *T̂*_*n*_ excitations and “disconnected” lower-level excitations. Critically, all excitations above the order *n* are approximated through the “disconnected” excitations. This property also gives coupled-cluster methods superior convergence to the FCI limit compared to their corresponding truncated CI counterpart, evidenced by their excellent agreement with experimental results for energetics,^5–10,15–17^ equilibrium geometries and spectroscopic properties,^18–24^ as well as magnetic properties.^25–28^

A truncated coupled-cluster computation scales as $O\left(n^{2n+2}\right)$ , where *n* represents the max excitation order treated directly with “connected” excitations. Hence, the CCSDTQ method scales as $O\left(n^{10}\right)$ with respect to the number of atoms *n*. Usually, excitations up to triples need to be considered to obtain results of chemical accuracy (<1 kcal mol^–1^ deviation from experiment for relative energies).^15,29–33^ The cost of treating triples exactly scales as $O\left(n^{8}\right)$ , leading to the development of the simpler CCSD­(T) method.^34,35^ CCSD­(T) involves estimation of triple excitations through perturbation theory from converged *T*_2_ and *T*_1_ CCSD amplitudes. The “relatively” cheaper $O\left(n^{7}\right)$ cost of CCSD­(T), combined with its ∼1 kcal mol^–1^ accuracy, led to it being dubbed the “gold-standard” method of electronic structure theory.^36–38^ In some cases, CCSD­(T) provides more accurate results compared to CCSDT due to a favorable error cancellation, which arises from the neglect of higher-order triples contributions.^37–41^ However, in applications where subchemical accuracy (<1 kJ mol^–1^) is required, including molecules with significant multireference character, CCSD­(T) does not necessarily yield reliable results.^42,43^ In mild to moderate cases, evidence suggests that the inclusion of higher-level excitations in single-reference coupled-cluster theory is able to account for multireference effects.^44–47^ The possibility of achieving such high levels of accuracy allows theoretical models to play a larger role in making novel chemical predictions. This is especially useful for the determination of accurate reaction enthalpies and barrier heights,^48–51^ where CCSDTQ gives “essentially exact” results.^52^

The $O\left(n^{10}\right)$ cost of the CCSDTQ method makes it intractable for most systems with more than 4–5 heavy atoms in a double-ζ basis set, given typical available methods and computing resources. Because of this, many approximations have been developed for CCSDTQ.^31,53–59^ One noteworthy approximation involves the use of perturbation theory, where by analogy to CCSDT and CCSD­(T), Bomble, Stanton, and coworkers devised the $O\left(n^{9}\right)$ CCSDT­(Q) method by adding back quadruples-like contributions from fifth- and sixth-order terms in perturbation theory.^55^ The (Q) correction, on average, accounts for around 90% of the difference between CCSDTQ and CCSDT in terms of absolute energies,^60^ and typically agrees with CCSDTQ to within 0.01 kcal mol^–1^ with regard to relative energies in many, but not all, cases.^37,38,40^ Due to its superb accuracy, CCSDT­(Q) was coined the “platinum standard”^52^ method of quantum chemistry. Another noteworthy approximation is the CCSDT­(Q)_Λ_ approach,^56^ which makes additional use of the “de-excitation” operator Λ, normally used to compute coupled-cluster derivatives or properties.^61,62^

Unfortunately, the high polynomial scaling of these algorithms fundamentally limits their applicability to larger molecules. Theoretically, linear-scaling electron correlation algorithms are possible due to the *R* ^–6^ nature of dynamic electron correlation. These have recently been achieved through orbital transformation or “local” approximations, including density-fitting (DF)/resolution of the identity (RI),^63–65^ rank reduction (RR),^66–70^ tensor hypercontraction (THC),^71–76^ cluster-in- molecule (CIM),^77–80^ projected atomic orbital (PAO),^81–86^ optimized virtual orbital (OVO),^87^ frozen natural orbital (FNO),^88,89^ local natural orbital (LNO),^90–93^ as well as pair natural orbital (PNO) approaches.^94–109^

Among those approximations, pair natural orbital (PNO)-based coupled-cluster methods have been the most popular because of the combination of their accuracy, tractability for larger molecules, and systematic convergence with their canonical counterparts. An example of such a method is the domain-based local pair natural orbital (DLPNO) approach of Neese and coworkers,^96,97,102,105^ which is known for the formation of PNOs through sparse projected atomic orbitals (PAOs) through a “cascaded” approach.^110^ They have successfully implemented DLPNO-based coupled-cluster methods through perturbative triples excitations, with DLPNO-CCSD­(T).^102,105^

Recently, we have derived, developed, and implemented the domain-based local pair natural orbital approach for both full triple [DLPNO-CCSDT][Bibr ref108] and perturbative quadruple excitations [DLPNO-CCSDT­(Q)][Bibr ref109] methods. Our DLPNO-CCSDT­(Q) work represents, to our knowledge, the first linear-scaling treatment of coupled-cluster quadruples through the use of local pair natural orbitals (PNOs). Among local correlation approaches, our work follows prior work by Rolik and Kállay with the local natural orbital (LNO)-based general-order coupled-cluster methods, including LNO–CCSDT­(Q) and CCSDTQ, available in the MRCC package.^90^ A linear-scaling framework has also been applied and extended to these methods,^111^ and recently, LNO–CCSDT­(Q) and LNO–CCSDTQ have also been extensively benchmarked for accuracy.^112^ DLPNO–CCSDT­(Q) is efficient and accurate, capturing (Q) contributions to within 0.01 kcal mol^–1^ with sufficiently tight parameters, and can handle larger molecules such as water clusters up to 49 molecules and 2842 basis functions with a triple-ζ basis set.^109^ However, in some cases, CCSDT­(Q) is known to overestimate the FCI correlation energy.^113^ This is especially problematic for molecular systems with strong multireference character such as Criegee intermediates,^114^ or in situations involving low energetic barriers such as with fulminic acid.^115^ Though CCSDT­(Q)_Λ_ can be a good alternative to CCSDT­(Q) for more difficult cases,^116^ a linear-scaling implementation of CCSDTQ represents a “holy grail” in modern computational quantum chemistry.

Here, we apply the DLPNO approach to full quadruples in the DLPNO–CCSDTQ approach. This is accomplished using quadruples amplitudes computed in the truncated quadruples natural orbitals (QNO) basis computed using DLPNO–CCSDT pair densities.^108^ This is similar to the approach used in our DLPNO–CCSDT­(Q) algorithm. Through this approach, we can practically match CCSDTQ-CCSDT­(Q) relative energy differences on the order of 0.01–0.05 kcal mol^–1^ for the systems studied. We also present, to the best of our knowledge, some of the largest CCSDTQ computations ever performed, with a benzene dimer as well as a large water cluster containing 17 molecules. Some of these larger computations may take thousands of years or longer if a canonical algorithm was utilized. Thus, our research represents a milestone in terms of approximating the FCI results for larger systems.

## 2 Theory

### 2.1 Notation

We use the following conventions to describe the indices of matrices and tensors appearing in this work, analogous to the notation used in our prior research:^107–109^

- μ, ν, λ, σ: atomic orbitals; these range from 1 to *n*_*bf*_, the number of basis functions
- *i*, *j*, *k*, *l*: canonical and local occupied molecular orbitals; these range from 1 to *n*_*occ*_, the number of occupied orbitals
- *a*, *b*, *c*, *d*: canonical virtual molecular orbitals; these range from 1 to *n*_*virt*_, the number of virtual orbitals
- *p*, *q*, *r*, *s*: general canonical molecular orbitals; these range from 1 to *n*_*occ*_ + *n*_*virt*_
- *μ̃*, *ν̃*, λ̃, *σ̃*: projected atomic orbitals; these range from 1 to *n*_*bf*_
- *μ̃*_*ij*_, *ν̃*_*ij*_, λ̃_*ij*_, *σ̃*_*ij*_: projected atomic orbitals localized to pair *ij*; these range from 1 to *n*_*pao*,*ij*_, the number of PAOs local to the LMO pair *ij*
- *μ̃*_*ijk*_, *ν̃*_*ijk*_, λ̃_*ijk*_, *σ̃*_*ijk*_: projected atomic orbitals localized to triple *ijk*; these range from 1 to *n*_*pao*,*ijk*_, the number of PAOs local to the LMO triple *ijk*
- *μ̃*_*ijkl*_, *ν̃*_*ijkl*_, λ̃_*ijkl*_, *σ̃*_*ijkl*_: projected atomic orbitals localized to quadruple *ijkl*; these range from 1 to *n*_*pao*,*ijkl*_, the number of PAOs local to the LMO quadruple *ijkl*
- *a*_*ij*_, *b*_*ij*_, *c*_*ij*_, *d*_*ij*_: Pair natural orbitals in each pair domain *ij*; these range from 1 to *n*_*pno*,*ij*_, the number of PNOs in the domain of the LMO pair *ij*
-
$\overset{\sim }{a_{ij}\;},\overset{\sim }{b_{ij}\;},\overset{\sim }{c_{ij}\;},\overset{\sim }{d_{ij}\;}$ : Extended pair natural orbitals in each pair domain *ij*; these range from 1 to *n*_*pno*,*ij* (ext)_, the number of XPNOs in the domain of the LMO pair *ij*
- *l*_*ijk*_, *m*_*ijk*_, *n*_*ijk*_: “Interacting” local molecular orbitals in a triples domain *ijk*, defined as the set of all occupied orbitals *l* such that *il*, *jl*, and *kl* are all strong or weak pairs; these range from 1 to *n*_*lmo*,*ijk*_, the number of LMOs in the domain of the LMO triplet *ijk*
- *a*_*ijk*_, *b*_*ijk*_, *c*_*ijk*_, *d*_*ijk*_: Triples natural orbitals in each triples domain *ijk*; these range from 1 to *n*_*tno*,*ijk*_, the number of TNOs in the domain of the LMO triplet *ijk*
- *m*_*ijkl*_, *n*_*ijkl*_: “Interacting” local molecular orbitals in a quadruples domain *ijkl*, defined as the set of all occupied orbitals *m* such that *im*, *jm*, *km*, and *lm* are all strong or weak pairs; these range from 1 to *n*_*lmo*,*ijkl*_,
- *a*_*ijkl*_, *b*_*ijkl*_, *c*_*ijkl*_, *d*_*ijkl*_: Quadruples natural orbitals in each quadruples domain *ijkl*; these range from 1 to *n*_*qno*,*ijkl*_, the number of QNOs in the domain of the LMO quadruplet *ijkl*
- *P*, *Q*: auxiliary basis functions for density-fitted ERIs; these range from 1 to *n*_*aux*_, the number of auxiliary basis functions for density fitting
- *P*_*ij*_, *Q*_*ij*_: local auxiliary basis functions in each pair domain *ij*; these range from 1 to *n*_*aux*,*ij*_, the number of auxiliary basis functions local to the LMO pair *ij*
- *P*_*ijk*_, *Q*_*ijk*_: local auxiliary basis functions in each triples domain *ijk*; these range from 1 to *n*_*aux*,*ijk*_, the number of auxiliary basis functions local to the LMO triplet *ijk*
- *P*_*ijkl*_, *Q*_*ijkl*_: local auxiliary basis functions in each quadruples domain *ijkl*; these range from 1 to *n*_*aux*,*ijkl*_, the number of auxiliary basis functions local to the LMO quadruplet *ijkl*

The relative sizes of these indices are usually:

4 $$\begin{array}{r} n_{qno,ijkl}<n_{pno,ij}<n_{tno,ijk}<n_{lmo,ij}<n_{lmo,ijk}<n_{lmo,ijkl}\ll n_{pao,ij}<n_{pao,ijk}<n_{pao,ijkl}\\ <n_{aux,ij}<n_{aux,ijk}<n_{aux,ijkl}∼O\left(1\right) \end{array}$$

5 $$n_{occ}\ll n_{virt}<n_{bf}<n_{naux}∼O\left(N\right)$$

where *N* is the system size represented by the number of atoms or electrons. The ordering *n*_*qno*,*ijkl*_ < *n*_*pno*,*ij*_ < *n*_*tno*,*ijk*_ occurs since the natural orbital cutoffs for quadruples amplitudes are not as tight as what is needed for doubles or triples amplitudes. The sizes of these local domains are determined by preset thresholds, where tighter thresholds yield larger domains with less error relative to the canonical method, with speed trading for accuracy.

### 2.2 CCSDTQ Working Equations

We model our working equations from the prior work of Matthews and Stanton^52^ and make use of the density-fitted *t*_1_-dressed formalism of DePrince and Sherrill to simplify the working equations, following our prior work.^63,107,108,117^ In this formalism, two-electron integrals are approximated with density-fitting/resolution of the identity^118–126^

6 $$\left(pq|rs\right)\approx \left(pq|P\right){\left(P|Q\right)}^{-1}\left(Q|rs\right)$$

This can also be written using the intermediate

7 $$B_{pq}^{Q}={\left(Q|P\right)}^{-\frac{1}{2}}\left(P|pq\right)$$

with

8 $$\left(pq|rs\right)\approx B_{pq}^{Q}B_{rs}^{Q}$$

Note that a very similar form can be achieved through Cholesky decomposition (CD).^127–129^ In the *t*_1_-dressed DF/CD formalism,^63,103,107^ the dressed integrals take the form, with the singles amplitudes $t_{i}^{a}$ folded into the integrals:

9 $${\mathrm{B̃}}_{ki}^{Q}=B_{ki}^{Q}+B_{ka}^{Q}t_{i}^{a}$$

10 $${\mathrm{B̃}}_{ia}^{Q}=B_{ia}^{Q}$$

11 $${\mathrm{B̃}}_{ai}^{Q}=B_{ai}^{Q}-t_{k}^{a}B_{ki}^{Q}+B_{ab}^{Q}t_{i}^{b}-t_{k}^{a}B_{kb}^{Q}t_{i}^{b}$$

12 $${\mathrm{B̃}}_{ab}^{Q}=B_{ab}^{Q}-t_{k}^{a}B_{kb}^{Q}$$

The *T*_1_-dressed Fock matrices are defined as^63^

13 $${\mathrm{F̃}}_{rs}={\mathrm{h̃}}_{rs}+\underset{Q,i}{\sum }2{\mathrm{B̃}}_{rs}^{Q}{\mathrm{B̃}}_{ii}^{Q}-{\mathrm{B̃}}_{ri}^{Q}{\mathrm{B̃}}_{is}^{Q}$$

with the *T*_1_-dressed one-electron Hamiltonian (*h̃*_*rs*_) defined with analogous rules as the density-fitted integrals. These dressed Fock matrices can also be written as^103,107^

14 $${\mathrm{F̃}}_{ki}={\mathrm{F̅}}_{ki}+{\mathrm{F̅}}_{ka}t_{i}^{a}$$

15 $${\mathrm{F̃}}_{ia}={\mathrm{F̅}}_{ia}$$

16 $${\mathrm{F̃}}_{ai}={\mathrm{F̅}}_{ai}-t_{k}^{a}{\mathrm{F̅}}_{ki}+{\mathrm{F̅}}_{ab}t_{i}^{b}-t_{k}^{a}{\mathrm{F̅}}_{kb}t_{i}^{b}$$

17 $${\mathrm{F̃}}_{ab}={\mathrm{F̅}}_{ab}-t_{k}^{a}{\mathrm{F̅}}_{kb}$$

where

18 $${\mathrm{F̅}}_{rs}=F_{rs}+\left[2\left(rs|kc\right)-\left(rc|ks\right)\right]t_{k}^{c}$$

This latter form of the *T*_1_-dressed Fock matrices will be used for our working equations due to explicit dependence solely on other Fock matrices. The singles, doubles, triples, and quadruples amplitudes are defined through the cluster operators *T*

19 $$T_{1}=t_{i}^{a}E_{ai}$$

20 $$T_{2}=\frac{1}{2}t_{ij}^{ab}E_{ai}E_{bj}$$

21 $$T_{3}=\frac{1}{6}t_{ijk}^{abc}E_{ai}E_{bj}E_{ck}$$

22 $$T_{4}=\frac{1}{24}t_{ijkl}^{abcd}E_{ai}E_{bj}E_{ck}E_{dl}$$

In spin-adapted, closed-shell coupled-cluster theory,

23 $$E_{ai}=a_{a}^{†}a_{i}+{\mathrm{a̅}}_{a}^{†}{\mathrm{a̅}}_{i}$$

The barred creation/annihilation operators refer to the β spin orbitals and the nonbarred ones refer to the α spin orbitals. In the *t*_1_-dressed formalism, the amplitudes are iterated to self-consistency through the solution of the following equation

24 $$R_{\mu }=\left\langle \Psi_{\mu }\left|e^{-T_{2}-T_{3}-T_{4}}\mathrm{H̃}e^{T_{2}+T_{3}+T_{4}}\right|\Psi_{0}\right\rangle$$

therein, Φ_*u*_ represents any excited determinant (up to quadruples) from the reference HF determinant, and *H̃* represents the *t*_1_-dressed Hamiltonian

25 $$\mathrm{H̃}=e^{-T_{1}}Ĥe^{T_{1}}$$

The amplitudes are iterated to self-consistency using the values of *R*_μ_, of which one simple approach is

26 $$t_{\mu }-=\frac{R_{\mu }}{\Delta_{\mu }}$$

where Δ_μ_ is the energy denominator corresponding to the excited determinant |Ψ_μ_⟩. For the excited determinant $\left|\Psi_{i...}^{a...}\right\rangle$ ,

27 $$\Delta_{i\cdot \cdot \cdot }^{a\cdot \cdot \cdot }=\epsilon_{a}+\cdot \cdot \cdot -\epsilon_{i}-\cdot \cdot \cdot$$

If noncanonical HF orbitals are used, the orbital energies (ϵ) are replaced by diagonal Fock matrix elements. We now define some “spin-adapted” intermediates involving the amplitudes.

28 $$U_{ij}^{ab}=2T_{ij}^{ab}-T_{ij}^{ba}$$

29 $$Z_{ijk}^{abc}=2T_{ijk}^{abc}-T_{ijk}^{bac}-T_{ijk}^{cba}$$

30 $$\alpha_{ijkl}^{abcd}=2T_{ijkl}^{abcd}-T_{ijkl}^{bacd}-T_{ijkl}^{cbad}-T_{ijkl}^{dbca}$$

31 $$\beta_{ijkl}^{abcd}=2\alpha_{ijkl}^{abcd}-\alpha_{ijkl}^{acbd}-\alpha_{ijkl}^{adcb}$$

These quantities are equivalent to $T_{ǐj}^{ǎb}$ , $T_{ǐjk}^{ǎbc}$ , $T_{ǐjkl}^{ǎbcd}$ , and $T_{ǐǰkl}^{ǎ\mathrm{b̌}cd}$ from the formalism of Matthews and Stanton.^52^ Next, we present the working equations for each of the residuals in CCSDTQ. Since quadruply excited determinants do not directly contribute to singly excited determinants, the equation for $R_{i}^{a}$ in CCSDTQ is the same as in CCSDT. The full set of working equations for these can be found in our prior work:^107,108^

32 $$R_{i}^{a}=R_{i}^{a}\left[\mathrm{CCSDT}\right]$$

The quadruples contribution to the doubles residual takes the form

33 $$R_{ij}^{ab}=R_{ij}^{ab}\left[\mathrm{CCSDT}\right]+P_{ij}^{ab}\left[\frac{1}{4}B_{me}^{Q}B_{nf}^{Q}\beta_{mnij}^{efab}\right]$$

where $P_{ij}^{ab}$ represents the permutation operator

34 $$P_{ij}^{ab}X_{ij}^{ab}=X_{ij}^{ab}+X_{ji}^{ba}$$

For the quadruples contribution to the triples residual,

35 $$R_{ijk}^{abc}=R_{ijk}^{abc}\left[\mathrm{CCSDT}\right]+P_{ijk}^{abc}\left[\frac{1}{6}{\mathrm{F̃}}_{me}\alpha_{mijk}^{eabc}+\frac{1}{2}{\mathrm{B̃}}_{ae}^{Q}B_{mf}^{Q}\alpha_{mijk}^{febc}-\frac{1}{2}B_{me}^{Q}{\mathrm{B̃}}_{nj}^{Q}\alpha_{mink}^{eabc}\right]$$

with

36 $$P_{ijk}^{abc}X_{ijk}^{abc}=X_{ijk}^{abc}+X_{ikj}^{acb}+X_{jik}^{bac}+X_{jki}^{bca}+X_{kij}^{cab}+X_{kji}^{cba}$$

The quadruple residual is significantly more complex, involving many intermediates:

37 $$\begin{array}{r} A_{ej}^{ab}={\mathrm{B̃}}_{ae}^{Q}{\mathrm{B̃}}_{bj}^{Q}+B_{me}^{Q}{\mathrm{B̃}}_{nj}^{Q}T_{mn}^{ab}+\frac{1}{2}\left[2B_{mf}^{Q}{\mathrm{B̃}}_{ae}^{Q}-{\mathrm{B̃}}_{af}^{Q}B_{me}^{Q}\right]U_{mj}^{fb}-\left(\frac{1}{2}+P_{ab}\right)\left[B_{me}^{Q}{\mathrm{B̃}}_{af}^{Q}T_{jm}^{fb}\right]\\ -B_{me}^{Q}B_{nf}^{Q}Z_{nmj}^{fab}-{\mathrm{F̃}}_{me}T_{mj}^{ab} \end{array}$$

38 $$\begin{array}{r} B_{ij}^{am}={\mathrm{B̃}}_{ai}^{Q}{\mathrm{B̃}}_{mj}^{Q}+{\mathrm{B̃}}_{ae}^{Q}B_{mf}^{Q}T_{ij}^{ef}+\frac{1}{2}\left[2B_{ne}^{Q}{\mathrm{B̃}}_{mj}^{Q}-{\mathrm{B̃}}_{nj}^{Q}B_{me}^{Q}\right]U_{ni}^{ea}-\left(\frac{1}{2}+P_{ij}\right)\left[{\mathrm{B̃}}_{nj}^{Q}B_{me}^{Q}T_{in}^{ea}\right]\\ +{\mathrm{F̃}}_{me}T_{ij}^{ae}+B_{me}^{Q}B_{nf}^{Q}Z_{nij}^{fae} \end{array}$$

39 $${\overset{\approx }{F}}_{ae}={\mathrm{F̃}}_{ae}-\left(2B_{nf}^{Q}B_{me}^{Q}-B_{ne}^{Q}B_{mf}^{Q}\right)T_{nm}^{fa}$$

40 $${\overset{\approx }{F}}_{mi}={\mathrm{F̃}}_{mi}+\left(2B_{nf}^{Q}B_{me}^{Q}-B_{ne}^{Q}B_{mf}^{Q}\right)T_{ni}^{fe}$$

41 $$E_{ei}^{ma}=\left[2B_{me}^{Q}{\mathrm{B̃}}_{ai}^{Q}-{\mathrm{B̃}}_{mi}^{Q}{\mathrm{B̃}}_{ae}^{Q}\right]+\left[2B_{nf}^{Q}B_{me}^{Q}-B_{ne}^{Q}B_{mf}^{Q}\right]U_{ni}^{fa}$$

42 $$F_{ie}^{ma}={\mathrm{B̃}}_{mi}^{Q}{\mathrm{B̃}}_{ae}^{Q}-B_{ne}^{Q}B_{mf}^{Q}T_{in}^{fa}$$

43 $$G_{ij}^{mn}={\mathrm{B̃}}_{mi}^{Q}{\mathrm{B̃}}_{nj}^{Q}+B_{me}^{Q}B_{nf}^{Q}T_{ij}^{ef}$$

44 $$H_{ef}^{ab}={\mathrm{B̃}}_{ae}^{Q}{\mathrm{B̃}}_{bf}^{Q}+B_{me}^{Q}B_{nf}^{Q}T_{mn}^{ab}$$

45 $$I_{eij}^{mab}=P_{ij}^{ab}\left[\left(2B_{me}^{Q}{\mathrm{B̃}}_{af}^{Q}-B_{mf}^{Q}{\mathrm{B̃}}_{ae}^{Q}\right)T_{ji}^{bf}-\left(2B_{me}^{Q}{\mathrm{B̃}}_{ni}^{Q}-{\mathrm{B̃}}_{mi}^{Q}B_{ne}^{Q}\right)T_{nj}^{ab}+\frac{1}{4}\left(2B_{nf}^{Q}B_{me}^{Q}-B_{ne}^{Q}B_{mf}^{Q}\right)Z_{nij}^{fab}\right]$$

46 $$J_{iej}^{mab}=B_{mf}^{Q}{\mathrm{B̃}}_{ae}^{Q}T_{ji}^{bf}-{\mathrm{B̃}}_{mi}^{Q}B_{ne}^{Q}T_{nj}^{ab}-\frac{1}{2}B_{ne}^{Q}B_{mf}^{Q}T_{inj}^{fab}$$

47 $$K_{ijk}^{amn}=P_{jk}^{mn}\left[B_{me}^{Q}{\mathrm{B̃}}_{nk}^{Q}T_{ij}^{ae}+\frac{1}{2}B_{me}^{Q}B_{nf}^{Q}T_{ijk}^{aef}\right]$$

48 $$L_{ijk}^{abm}=P_{ij}^{ab}\left[{\mathrm{B̃}}_{ae}^{Q}B_{mf}^{Q}T_{ijk}^{ebf}+\frac{1}{2}\left(E_{ei}^{ma}+F_{ie}^{ma}\right)T_{jk}^{be}+F_{ke}^{ma}T_{ji}^{be}-\frac{1}{2}G_{ki}^{mn}T_{nj}^{ab}+\frac{1}{2}B_{me}^{Q}B_{nf}^{Q}\alpha_{nijk}^{fabe}\right]$$

49 $$M_{ejk}^{abc}=P_{jk}^{bc}\left[\frac{1}{2}H_{ef}^{ab}T_{jk}^{fc}-\frac{1}{2}B_{me}^{Q}B_{nf}^{Q}\alpha_{nmjk}^{fabc}\right]$$

The operators *P*_*ij*_ and *P*_*ab*_ permute the indices of *i* and *j*; and *a* and *b*, respectively. The final form of the quadruples residual takes the succinct form

50 $$\begin{array}{ll} R_{ijkl}^{abcd} & =P_{ijkl}^{abcd}[\frac{1}{2}A_{ej}^{ab}T_{ikl}^{ecd}-\frac{1}{2}B_{ij}^{am}T_{mkl}^{bcd}+\frac{1}{6}{\overset{\approx }{F}}_{ae}T_{ijkl}^{ebcd}-\frac{1}{6}{\overset{\approx }{F}}_{mi}T_{mjkl}^{abcd}+\frac{1}{12}E_{ei}^{ma}\alpha_{mjkl}^{ebcd}\\ & -\frac{1}{2}\left(\frac{1}{2}+P_{ab}\right)F_{ie}^{ma}T_{jmkl}^{ebcd}+\frac{1}{4}G_{ij}^{mn}T_{mnkl}^{abcd}+\frac{1}{4}H_{ef}^{ab}T_{ijkl}^{efcd}+\frac{1}{8}I_{eij}^{mab}Z_{mkl}^{ecd}\\ & -\left(\frac{1}{2}+P_{ac}\right)J_{iej}^{mab}T_{kml}^{ecd}+\frac{1}{2}K_{ijk}^{amn}T_{mnl}^{bcd}-\frac{1}{2}L_{ijk}^{abm}T_{ml}^{cd}+\frac{1}{2}M_{ejk}^{abc}T_{il}^{ed}] \end{array}$$

with

51 $$P_{ijkl}^{abcd}X_{ijkl}^{abcd}=X_{ijkl}^{abcd}+X_{jikl}^{bacd}+X_{kjil}^{cbad}+...+X_{lkji}^{dcba}$$

where all the possible column symmetries are permuted over, analogous to $P_{ij}^{ab}$ and $P_{ijk}^{abc}$ . We note that this is, to our knowledge, one of the most compact presentations of the closed-shell spin-adapted CCSDTQ equations due to the use of the *t*_1_-dressed formalism.

## 3 Algorithmic Details

### 3.1 Domain-Based Local Pair Natural Orbital Methodology

#### 3.1.1 Local Molecular Orbitals (LMOs) and Prescreening

Local molecular orbitals (LMOs) are computed through a unitary transformation of the canonical Hartree–Fock molecular orbitals over the active occupied space.^130^

52 $$C_{\mu i}^{L}=\underset{j\in \mathrm{active occ}}{\sum }C_{\mu j}U_{ji}$$

The choice of *U* can be derived based on minimizing or maximizing certain properties of the transformed orbitals. Such methods include Foster–Boys,^131,132^ which minimizes spatial extent; Pipek–Mezey,^132,133^ maximizing atomic partial charges; as well as Edminston–Ruedenberg,^134,135^ maximizing orbital self-repulsion. In our prior work,^107–109^ we have utilized the Foster–Boys method, following the work of Riplinger et al.^102^ However, we have discovered that Pipek–Mezey orbitals yield slightly more compact natural orbital spaces for the corresponding localized MO pairs, triplets, and quadruplets. Thus, in this work we utilize Pipek–Mezey orbitals in all reported computations.

In the preceding DLPNO–CCSD computation,^102,107^ pairs of LMOs are first prescreened using a dipole estimate,^101^ and pairs that do not survive the initial dipole prescreening procedure (energies below *T*_CUT_PRE_ and with an overlap below the *T*_CUT_DO_IJ_) are denoted “dipole pairs”, and are not further considered, with their energy contribution accounted through the sum of their individual estimated dipole energies (Δ*E*_dipole_). This step is denoted in the “dipole estimate” section of Figure 1. Next, noniterative semicanonical local MP2 (SC-LMP2) energies are computed for each surviving pair. These are known as “semi-canonical” since they ignore the contribution of small off-diagonal LMO Fock matrix elements in a noncanonical HF representation. The SC-LMP2 energies are also computed in the larger projected atomic orbital (PAO) basis rather than the sparser pair natural orbital (PNO) basis, since these energy estimates are relatively inexpensive to compute. As shown in the “PAO-LMP2” section of Figure 1, pairs that have an energy contribution lower than *T*_CUT_PAIRS_MP2_ are denoted “semi-canonical MP2 pairs” and eliminated from consideration in the rest of the computation. The sum of their estimated SC-LMP2 energies is denoted as Δ*E*_SC‑LMP2_. After the dipole and semicanonical MP2 pairs are screened out, all surviving LMO pairs are used in further consideration for the rest of the computation. These pairs are subdivided into “weak pairs” and “strong pairs”, with the amplitudes of weak pairs being computed in the local MP2 basis, and not being further updated (although their coupling with strong pair doubles amplitudes, triples amplitudes, and quadruples amplitudes is still considered in DLPNO–CCSD, DLPNO–CCSDT, DLPNO–CCSDTQ). The cutoff to distinguish between weak pairs and strong pairs is *T*_CUT_PAIRS_.

**Figure 1:**
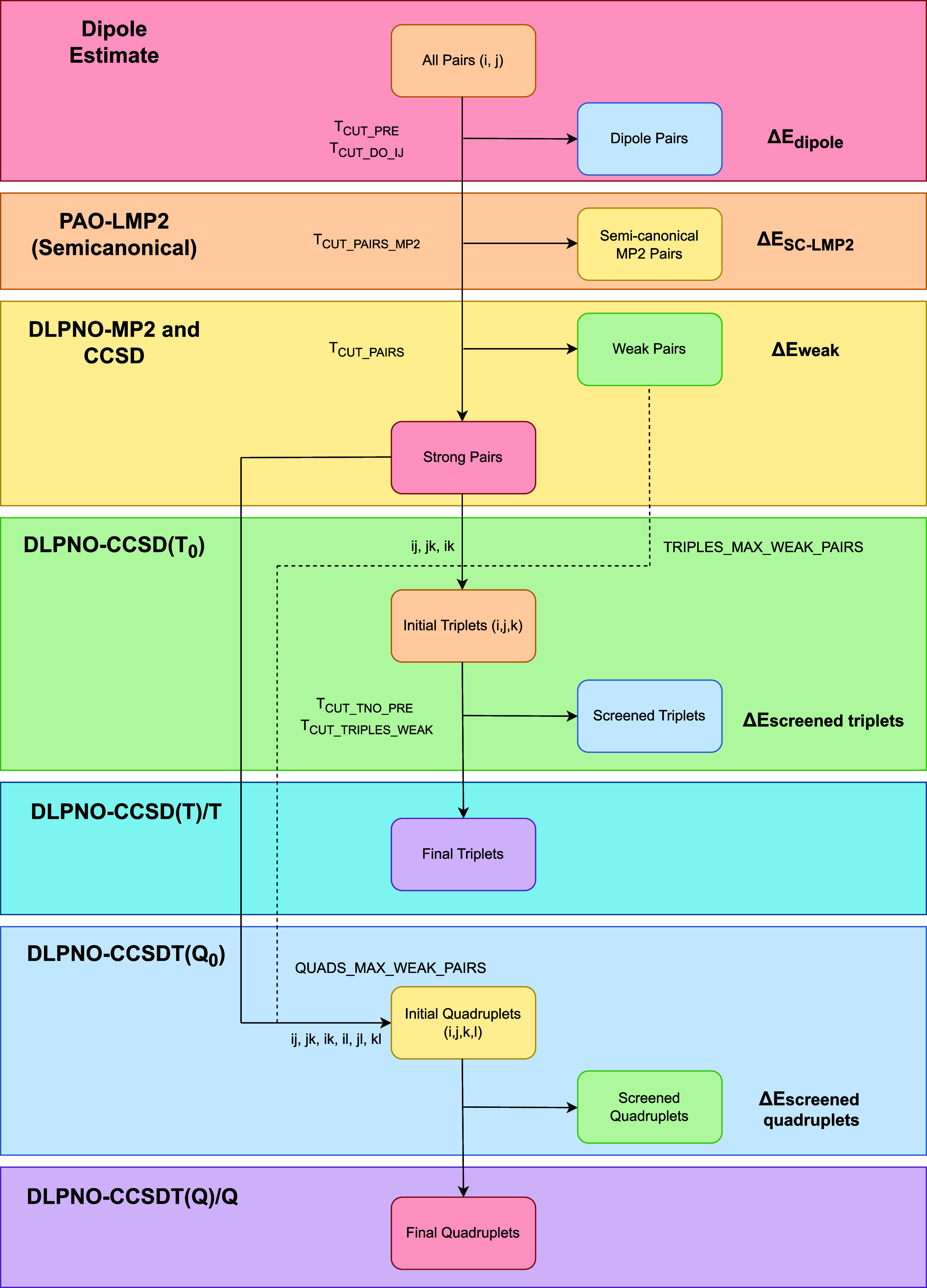
A flowchart representing all of the LMO-related prescreening that occurs through the course of a complete DLPNO–CCSDTQ computation, with the corresponding level of theory at which the prescreening occurs. An accounting of the energy contributions at each step is also given.

For the triples prescreening procedure, the initial subset of triplets (*i*, *j*, *k*) is determined from the union of pairs *ij*, *jk*, and *ik* such that they are strong or weak pairs.^97,105,107^ The number of weak pairs allowed (out of the three) can be limited through the parameter TRIPLES_MAX_WEAK_PAIRS. Due to the linear-scaling number of interacting pairs, the total number of interacting triplets will also be commensurate with the size of the system. After the initial list is determined, a semicanonical DLPNO-(T_0_) energy estimate is computed for each triplet, as shown in the “DLPNO–CCSD­(T_0_)” section in Figure 1. These can be computed using looser cutoffs (*T*_CUT_TNO_PRE_ for the triple natural orbitals) since only an estimate is required. The triples with an energy contribution less than *T*_CUT_TRIPLES_WEAK_ are not further considered in the computation, with the sum of their contributions being accounted for through Δ*E*_screened_triplets_. Amplitudes for all surviving triplets, $t_{ijk}^{abc}$ , are computed in full iterative DLPNO–CCSD­(T),^105,107^ as well as DLPNO–CCSDT.^108^

Following a DLPNO–CCSDT computation,^108^ initial quadruplets (*i*, *j*, *k*, *l*) are also determined from strong and weak pairs *ij*, *ik*, *il*, *jk*, *jl*, and *kl*. The maximum number of weak pairs allowed per quadruplet is set through the parameter QUADS_MAX_WEAK_PAIRS (out of a maximum of 6). The corresponding CCSDT doubles amplitudes from these pairs are used to form the quadruple natural orbital (QNO) space for quadruplet *ijkl*. Similar to triples, these quadruplets are prescreened using a semicanonical DLPNO-(Q_0_) estimate^109^ at a looser quadruples natural orbital (QNO) cutoff *T*_CUT_QNO_PRE_, with energy contribution Δ*E*_screened_quadruplets_, as shown in the “DLPNO–CCSDT­(Q_0_)” section of Figure 1. The energy cutoff for prescreening these quadruplets is *T*_CUT_QUADS_WEAK_. Since the corresponding DLPNO–CCSD computation is relatively inexpensive compared to DLPNO–CCSDT or DLPNO–CCSDTQ, and tighter accuracy is demanded for applications of higher-order coupled-cluster, we tighten *T*_CUT_PAIRS_ to 10^–8^ (from the default 10^–6^ used with VeryTightPNO convergence settings), to match the values of the *T*_CUT_TRIPLES_WEAK_ and *T*_CUT_QUADS_WEAK_ parameters. With all the prescreening performed, the overall DLPNO–CCSDTQ energy expression is as follows:

53 $$E_{\mathrm{DLPNO}-\mathrm{CCSDTQ}}=E_{\mathrm{LCCSDTQ}}+\left(\Delta E_{\mathrm{weak}}+\Delta E_{\mathrm{SC}-\mathrm{LMP}2}+\Delta E_{\mathrm{dipole}}\right)+\Delta E_{\mathrm{screened}\_\mathrm{triplets}}+\Delta E_{\mathrm{screened}\_\mathrm{quadruplets}}+\Delta E_{\mathrm{PNO}}$$

with all the different corrections from pre-screening of LMOs given in Figure 1. Δ*E*_PNO_ represents an estimated rank error over strong and weak pairs computed at the SC-LMP2 (energy loss incurred from going from PAOs to tighter PNOs used for full iterative LMP2) and PNO-LMP2 levels of theory (energy loss incurred from tighter PNOs used in full iterative LMP2 to the relatively looser PNOs used at the coupled-cluster level). These different virtual spaces are shown in more detail in Figure 2. *E*_LCCSDTQ_ represents the standard spin-adapted, closed-shell coupled-cluster energy expression using converged CCSDTQ singles and doubles amplitudes but in the local orbital basis:

**Figure 2:**
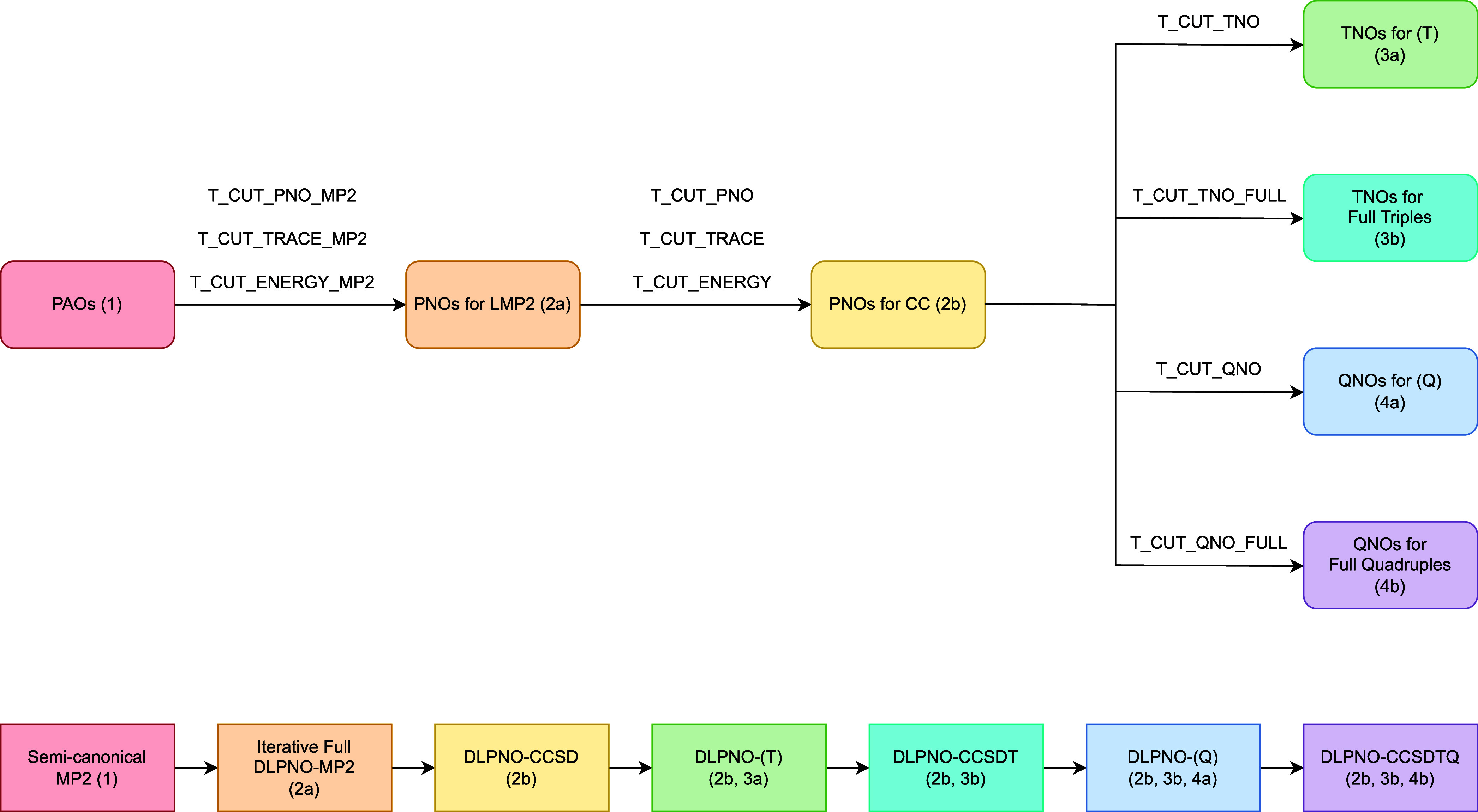
Depiction of the flow of information from the different virtual spaces in the DLPNO–CCSDTQ call pipeline; the methods are highlighted at the bottom, with each virtual orbital space used in each method given in parentheses.

54 $$E_{\mathrm{LCCSDTQ}}=\left[t_{ij}^{ab}+t_{i}^{a}t_{j}^{b}\right]\cdot \left[2\left(ia|jb\right)-\left(ib|ja\right)\right]$$

#### 3.1.2 Local Density-Fitting/RI Domains

Similar to our prior implementations of DLPNO with lower-order coupled-cluster methods,^107–109^ following the work of Neese and coworkers,^96,97,102^ atom-centered auxiliary functions for density fitting are assigned to pair, triples, and quadruples domains. This is done by computing the Mulliken population^136^ of electrons from LMO orbital *i* on each atomic center *A*

55 $$P_{\mu \nu }^{i}=C_{\mu i}^{L}S_{\mu \nu }C_{\nu i}^{L}$$

56 $$q_{iA}=2\underset{\mu \in A}{\sum }\underset{\nu }{\sum }P_{\mu \nu }^{i}\cdot \frac{P_{\mu \mu }^{i}}{P_{\mu \mu }^{i}+P_{\nu \nu }^{i}}$$

For each LMO *i*, if the Mulliken population on center *A* is higher than a given tolerance (*T*_CUT_MKN_), all of the auxiliary fitting functions centered on atom *A* are included in the local auxiliary domain of LMO *i*. The corresponding local auxiliary domain for pairs *ij* (functions *P*_*ij*_, *Q*_*ij*_) is formed from the union of the auxiliary domain of LMOs *i* and *j*. For triples (*P*_*ijk*_, *Q*_*ijk*_), the auxiliary domain is formed from the union of the auxiliary domains of its constituent LMOs *i*, *j*, and *k*. For quadruples (*P*_*ijkl*_, *Q*_*ijkl*_), the domains are analogously formed through the union of functions from *i*, *j*, *k*, and *l*. We note that the cutoffs used for triples and quadruples are not as tight as the ones used for pairs, since they include contributions from multiple LMO domains, and the error introduced by these approximations is not larger than the difference between the canonical density-fitted CCSDTQ correlation energy and canonical nondensity-fitted CCSDTQ (error on the order of 10^–5^ Eh).

#### 3.1.3 Projected Atomic Orbitals (PAOs)

Projected atomic orbitals (PAOs)^81^ can be used as a basis for the virtual space of a molecular system by the subtraction of the span of the occupied orbital space from the complete atomic orbital (AO)/molecular orbital (MO) space:

57 $$C_{\mu \mathrm{\nu ̃}}^{\mathrm{PAO}}=\delta_{\mu \nu }-C_{\mu i}^{\mathrm{MO}}C_{\sigma i}^{\mathrm{MO}}S_{\sigma \nu }^{\mathrm{AO}}$$

58 $$S_{\mathrm{\mu ̃}\mathrm{\nu ̃}}^{\mathrm{PAO}}=C_{\lambda \mathrm{\mu ̃}}^{\mathrm{PAO}}S_{\lambda \sigma }^{\mathrm{AO}}C_{\sigma \mathrm{\nu ̃}}^{\mathrm{PAO}}$$

Thus, PAOs (*ν̃*) can be expressed as a linear combination of atomic orbitals (μ) through the coefficient matrix *C* ^PAO^, and their overlap (*S* ^PAO^) can be computed according to the AO overlap matrix. Note that it is important to normalize the PAOs after computation to ensure that ⟨*μ̃*|*μ̃*⟩ = 1 for all PAOs μ.

59 $$C_{\mu \mathrm{\nu ̃}}^{\mathrm{PAO}}\leftarrow {\left(S_{\mathrm{\nu ̃}\mathrm{\nu ̃}}^{\mathrm{PAO}}\right)}^{-\frac{1}{2}}C_{\mu \mathrm{\nu ̃}}^{\mathrm{PAO}}$$

Though PAOs span the complete virtual space of the molecule, they are linearly dependent, being a rank *n*_*bf*_ basis for a space of rank *n*_*bf*_ – *n*_*occ*_. Nonetheless, PAOs were used in the earliest generation of local correlation methods,^82–86^ due to the sparsity of the AO density matrix as well as the AO overlap matrix. This can be shown by applying the relation $D_{\mu \sigma }^{\mathrm{AO}}=C_{\mu i}^{\mathrm{MO}}C_{\sigma i}^{\mathrm{MO}}$ to eq 57:

60 $$C_{\mu \mathrm{\nu ̃}}^{\mathrm{PAO}}=\delta_{\mu \nu }-D_{\mu \sigma }^{AO}S_{\sigma \nu }^{\mathrm{AO}}$$

For clarity, we note that the canonical virtual orbitals, unlike canonical occupied orbitals, are not required in this procedure. This is because any orthogonal complement of the occupied space in the complete MO space forms a valid basis for virtual orbitals with PAOs satisfying that criterion by construction. In these early local correlation methods, PAOs are first assigned to an LMO domain (e.g., *ij*) through some measure of sparsity, such as atomic separation or using a cutoff distance. In our code, following the work of Pinski, Riplinger, Valeev, and Neese,^101^ a grid-based “differential overlap integral” (DOI) between an LMO *i* and a PAO *μ̃* is computed as a measure for sparsity, inspired by atomic orbital ERI screening techniques:^137^

61 $${\mathrm{DOI}}_{i\mathrm{\mu ̃}}={\left(\int |\phi_{i}\left(r\right){|}^{2}|\phi_{\mathrm{\mu ̃}}\left(r\right){|}^{2}dr\right)}^{1/2}$$

The PAO *μ̃* is then assigned to the domain of LMO *i* if the value of the integral exceeds the *T*_DO_ parameter. Thus, the PAO domain of pair *μ̃*_*ij*_ of *ij* is formed from the union of all PAOs *μ̃*_*i*_ in the domain of LMO *i* and *μ̃*_*j*_ in the domain of LMO *j*. After the initial assignment of PAOs to the relevant pair domains, they can be transformed into an orthogonal basis through Partial Cholesky decomposition (other suitable techniques include Gram–Schmidt or Löwdin):

62 $$\left|{\mathrm{\mu ̃}}_{ij}^{\prime }\right\rangle =X_{{\mathrm{\nu ̃}}_{ij}{\mathrm{\mu ̃}}_{ij}^{\prime }}^{\mathrm{orthog}}\left|{\mathrm{\nu ̃}}_{ij}\right\rangle$$

where the transformation matrix **X** ^orthog^ is obtained from the given choice of orthogonalization algorithm, in the case of Löwdin orthogonalization:

63 $$X_{{\mathrm{\nu ̃}}_{ij}{\mathrm{\mu ̃}}_{ij}^{\prime }}^{\mathrm{orthog}}=U_{{\mathrm{\nu ̃}}_{ij}{\mathrm{\mu ̃}}_{ij}^{\prime }}^{\mathrm{svd}}\sigma_{{\mathrm{\mu ̃}}_{ij}^{\prime }}^{-1/2}$$

where $\sigma_{{\mathrm{\mu ̃}}_{ij}^{\prime }}$ represents the truncated singular values of the initial PAO overlap matrix $S_{{\mathrm{\nu ̃}}_{ij}{\mathrm{\nu ̃}}_{ij}}$ , where all values below a certain tolerance (e.g., 10^–8^) are removed. Next, a Fock matrix is computed on the transformed PAO basis:

64 $$F_{{\mathrm{\mu ̃}}_{ij}^{\prime }{\mathrm{\nu ̃}}_{ij}^{\prime }}=X_{{\mathrm{\mu ̃}}_{ij}{\mathrm{\mu ̃}}_{ij}^{\prime }}^{\mathrm{orthog}}F_{{\mathrm{\mu ̃}}_{ij}{\mathrm{\nu ̃}}_{ij}}X_{{\mathrm{\nu ̃}}_{ij}{\mathrm{\nu ̃}}_{ij}^{\prime }}^{\mathrm{orthog}}$$

The Fock matrix in the transformed PAO basis is then diagonalized to make a canonical virtual space (i.e., no off-diagonal Fock matrix elements), with the eigenvalues $\epsilon_{{\mathrm{\mu ̃}}_{ij}^{\prime \prime }}$ representing “orbital energies” and the set of “canonicalized PAOs” defined as

65 $$\left|{\mathrm{\mu ̃}}_{ij}^{\prime \prime }\right\rangle =X_{{\mathrm{\mu ̃}}_{ij}{\mathrm{\mu ̃}}_{ij}^{\prime }}^{\mathrm{orthog}}U_{{\mathrm{\mu ̃}}_{ij}^{\prime }{\mathrm{\mu ̃}}_{ij}^{\prime \prime }}^{\mathrm{diag}}\left|{\mathrm{\mu ̃}}_{ij}\right\rangle =X_{{\mathrm{\mu ̃}}_{ij}{\mathrm{\mu ̃}}_{ij}^{\prime \prime }}^{\mathrm{canon}}\left|\mu_{ij}\right\rangle$$

where $U_{{\mathrm{\mu ̃}}_{ij}^{\prime }{\mathrm{\mu ̃}}_{ij}^{\prime \prime }}^{\mathrm{diag}}$ represents the eigenvectors of $F_{{\mathrm{\mu ̃}}_{ij}^{\prime }{\mathrm{\nu ̃}}_{ij}^{\prime }}$ .

The mutual sparsity of LMOs/PAOs ensures that each LMO *i* and LMO pair *ij* will have asymptotically a constant number of PAOs with increasing system size, giving these early LMO/PAO-based local correlation methods asymptotic linear scaling. However, there is often a high crossover point with PAO-based local correlation methods and canonical correlated methods, and more modern local correlation methods are based on using pair natural orbitals (PNOs). However, PAOs are still used as intermediates in many PNO-based methods today,^92,96,103,107^ notably in the domain-based local pair natural orbital (DLPNO) family of methods,^96,97,101,102,107–109^ to which our latest algorithm belongs. In such methods, sparse, truncated PAOs are used to form an initial virtual basis to express density-fitted ERIs before they are transformed to the PNO basis. This method of computing PNOs from sparse PAOs rather than canonical MOs is known as the “cascaded” approach, allowing for linear-scaling computation of necessary ERIs, and circumvents the need of using nonsparse canonical MOs altogether.^96,102^ For triplets *ijk*, the redundant PAOs are similarly assigned through the union of PAOs assigned through differential overlap from their constituent LMOs *i*, *j*, and *k*, before they are canonicalized to form $\left|{\mathrm{\mu ̃}}_{ijk}^{\prime \prime }\right\rangle$ . These canonicalized triplet PAO domains are used as an initial basis to compute triplet natural orbitals (TNOs). For quadruples, the same procedure applies, where the initial redundant PAOs come through the union of PAOs from *i*, *j*, *k*, and *l* before canonicalization $\left(\left|{\mathrm{\mu ̃}}_{ijkl}^{\prime \prime }\right\rangle \right)$ , forming the initial basis for quadruples natural orbitals (QNOs).

#### 3.1.4 Pair, Triples, and Quadruples Natural Orbitals (PNOs, TNOs, QNOs)

Pair natural orbitals were known in the early days of computational quantum chemistry as “pseudonatural orbitals” following the work of Edmiston and Krauss in 1965,^138^ around two decades before the introduction of projected atomic orbitals (PAOs) by Pulay in 1983.^81^ PNOs saw popular use in the early 1970s with the early PNO-based configuration interaction (PNO–CI)^139–141^ and independent electron pair approximation-based PNO (IEPA-PNO) methods.^142^ However, the rise in popularity of the more efficient direct CI approaches^143^ led to a decline in the popularity of PNO-based algorithms. With the advent of pair-domain-based local correlation methods in the 1990s and early 2000s,^81,82,84^ Neese and coworkers reintroduced PNOs in quantum chemistry as an alternate basis to express localized virtual orbitals, as opposed to projected atomic orbitals (PAOs), with their seminal work on their local pair natural orbital (LPNO)-CEPA^94^ and LPNO–CCSD^95^ methods. PNOs are a much more compact representation of the virtual orbital space compared with PAOs, making PNO-based local correlation methods orders of magnitude faster than their PAO-based counterparts. PNOs are defined to be the eigenvectors of the pair density matrix **D**_**ij**_ of LMO pair *ij*

66 $$D_{ij}^{ab}=\frac{1}{1+\delta_{ij}}\left[u_{ij}^{ac}t_{ij}^{bc}+u_{ij}^{ca}t_{ij}^{cb}\right]$$

where $t_{ij}^{ab}$ represents the doubles amplitudes, and $u_{ij}^{ab}$ is defined as $2t_{ij}^{ab}-t_{ij}^{ba}$ . For pair natural orbitals preceding a CEPA or CCSD computation, these amplitudes are initially computed at the MP2 level of theory:^144^

67 $$t_{ij}^{ab}=\frac{\left(ia|jb\right)}{-\epsilon_{a}-\epsilon_{b}+\epsilon_{i}+\epsilon_{j}}$$

These can be computed using canonical virtual orbitals, canonicalized PAOs, or an initially larger PNO space. In the original LPNO-CEPA and LPNO–CCSD methods, Neese and coworkers initially utilized canonical virtual orbitals in the computation of pair natural orbitals.^94,95^ Unfortunately, this introduces the expensive $O\left(N^{5}\right)$ scaling of ERI transformations associated with canonical MP2 for these approaches. As mentioned in the previous section, Neese et al. pioneered the use of the “cascaded” approach in the formation of PNOs from local canonicalized PAOs in the updated domain-based local pair natural orbital (DLPNO) methodology.^96,97,102^ DLPNO also introduces the application of local auxiliary basis functions to approximate ERIs on the LMO/PNO basis. This makes the integral transformation steps more efficient as well as asymptotically linear-scaling. The initial set of PNOs computed in the LMO/PAO basis are known as *semicanonical* MP2 PNOs since eq 67 provides exact MP2 amplitudes only in the context of canonical Hartree–Fock molecular orbitals with no off-diagonal Fock matrix elements. The initial PNO transformation matrix from canonicalized PAOs takes the form:

68 $$D_{ij}^{{\mathrm{\mu ̃}}_{ij}^{\prime \prime }{\mathrm{\nu ̃}}_{ij}^{\prime \prime }}=X_{{\mathrm{\mu ̃}}_{ij}^{\prime \prime }a_{ij}}^{\mathrm{PNO},ij}n_{a_{ij}}^{occ,ij}X_{{\mathrm{\nu ̃}}_{ij}^{\prime \prime }a_{ij}}^{\mathrm{PNO},ij}$$

Similar to truncated SVD/eigenvalue-based rank reduction approaches,^145,146^ a new basis for the virtual space can be formed by the selection of the eigenvectors (the columns of $X_{{\mathrm{\mu ̃}}_{ij}^{\prime \prime }a_{ij}}^{\mathrm{PNO},ij}$ ) with corresponding eigenvalues $n_{a_{ij}}^{occ,ij}$ that are above a given tolerance (*T*_CUT_PNO_). These eigenvalues are also known as occupation numbers. Alternate approaches to select the eigenvectors include trace^107^ and energy criteria,^103,107^ which select the eigenvectors sorted in descending order of eigenvalue until the ratio of the sum of the selected eigenvalues to the sum of all the eigenvalues exceeds a certain threshold (*T*_CUT_TRACE_) or the MP2 energy computed using the truncated set divided by the energy computed in the full canonicalized PAO space exceeds *T*_CUT_ENERGY_. For the formation of PNOs in the preceding DLPNO-MP2 and DLPNO–CCSD steps in our computation, energy, and trace criterion are used since only single and double amplitudes directly contribute to the coupled-cluster correlation energy. For singles amplitudes $t_{i}^{a}$ , the virtual space is assigned from PNOs of diagonal pairs *ii*, with the diagonal pairs truncated at a tighter occupation number tolerance (*T*_CUT_PNO_ × *T*_DIAG_SCALE_). *T*_DIAG_SCALE_ is applied to all PNO-type cutoffs ( $T_{\mathrm{CUT}\_\mathrm{PNO}\_\mathrm{MP}2}$ , *T*_CUT_PNO_, *T*_CUT_XPNO_), and defaults to 3 × 10^–2^, following Neese and coworkers with the ORCA package.^147^ After the selection of PNOs, the PNOs are then canonicalized, similar to PAOs,

69 $$F_{a_{ij}b_{ij}}=X_{{\mathrm{\mu ̃}}_{ij}^{\prime \prime }a_{ij}}^{\mathrm{PNO},\mathrm{trunc}}F_{{\mathrm{\mu ̃}}_{ij}^{\prime \prime }{\mathrm{\nu ̃}}_{ij}^{\prime \prime }}^{\mathrm{PAO}}X_{{\mathrm{\nu ̃}}_{ij}^{\prime \prime }b_{ij}}^{\mathrm{PNO},\mathrm{trunc}}$$

70 $$\left|a_{ij}^{\prime }\right\rangle =U_{a_{ij}a_{ij}^{\prime }}^{\mathrm{diag}}\left|a_{ij}\right\rangle$$

where $U_{a_{ij}a_{ij}^{\prime }}^{\mathrm{diag}}$ represents the eigenvectors of $F_{a_{ij}b_{ij}}$ , and $X_{{\mathrm{\mu ̃}}_{ij}^{\prime \prime }a_{ij}}^{\mathrm{PNO},\mathrm{trunc}}$ is the PNO transformation matrix (eq 68), but with the truncated columns. Therefore, the final equation to transform from pair-localized redundant PAOs to canonicalized PAOs takes the following form:

71 $$X_{{\mathrm{\mu ̃}}_{ij}a_{ij}^{\prime }}^{\mathrm{PNO}}=X_{{\mathrm{\mu ̃}}_{ij}{\mathrm{\mu ̃}}_{ij}^{\prime \prime }}^{\mathrm{canon}}X_{{\mathrm{\mu ̃}}_{ij}^{\prime \prime }a_{ij}}^{\mathrm{PNO},\mathrm{trunc}}U_{a_{ij}a_{ij}^{\prime }}^{\mathrm{diag}}$$

$X_{{\mathrm{\mu ̃}}_{ij}{\mathrm{\mu ̃}}_{ij}^{\prime \prime }}^{\mathrm{canon}}$ represents the transformation from the pair-localized redundant PAOs to canonicalized PAOs (eq 65). Since the initial noncanonicalized PNOs are no longer needed elsewhere in the computation, the nonprimed *a*_*ij*_ will now represent canonicalized PNOs rather than the initial noncanonicalized PNOs in the rest of this work. Therefore, the overall transformation is

72 $$\left|a_{ij}\right\rangle =X_{{\mathrm{\mu ̃}}_{ij}a_{ij}}^{\mathrm{PNO}}\left|{\mathrm{\mu ̃}}_{ij}\right\rangle$$

In the case of triples, the corresponding triples natural orbitals (TNOs), labeled as |*a*_*ijk*_⟩, are analogously computed from the eigenvectors of the triples density, truncated at a tolerance *T*_CUT_TNO_. The triples density is defined as the average of the pair density of its corresponding three subpairs:^97^

73 $$D_{\mathrm{ijk}}=\frac{1}{3}\left(D_{\mathrm{ij}}+D_{\mathrm{jk}}+D_{\mathrm{ik}}\right)$$

The pair densities are computed from converged DLPNO–CCSD,^96,102,107^ doubles amplitudes in the pair natural orbital basis, then projected into the canonicalized PAO space for triplet *ijk*:

74 $$D_{ij}^{{\mathrm{\mu ̃}}_{ijk}^{\prime \prime }{\mathrm{\nu ̃}}_{ijk}^{\prime \prime }}=S_{a_{ij}}^{{\mathrm{\mu ̃}}_{ijk}^{\prime \prime }}D_{ij}^{a_{ij}b_{ij}}S_{b_{ij}}^{{\mathrm{\nu ̃}}_{ijk}^{\prime \prime }}$$

Above, $S_{a_{ij}}^{{\mathrm{\mu ̃}}_{ijk}^{\prime \prime }}$ is an overlap matrix computed through

75 $$S_{a_{ij}}^{{\mathrm{\mu ̃}}_{ijk}^{\prime \prime }}=X_{{\mathrm{\mu ̃}}_{ijk}{\mathrm{\mu ̃}}_{ijk}^{\prime \prime }}^{\mathrm{canon}}S_{{\mathrm{\nu ̃}}_{ij}}^{{\mathrm{\mu ̃}}_{ijk}}X_{{\mathrm{\nu ̃}}_{ij}a_{ij}}^{\mathrm{PNO}}$$

For quadruples, an analogous quadruples density **D**_**ijkl**_ is computed, using converged DLPNO–CCSDT^108^ doubles amplitudes:^109^

76 $$D_{\mathrm{ijkl}}=\frac{1}{6}\left(D_{\mathrm{ij}}+D_{\mathrm{ik}}+D_{\mathrm{il}}+D_{\mathrm{jk}}+D_{\mathrm{jl}}+D_{\mathrm{kl}}\right)$$

The quadruple density is computed from the DLPNO–CCSDT pair densities computed in the PNO space of each of its corresponding pairs projected onto the canonicalized PAO space of quadruples *ijkl*.

77 $$D_{ij}^{{\mathrm{\mu ̃}}_{ijkl}^{\prime \prime }{\mathrm{\nu ̃}}_{ijkl}^{\prime \prime }}=S_{a_{ij}}^{{\mathrm{\mu ̃}}_{ijkl}^{\prime \prime }}D_{ij}^{a_{ij}b_{ij}}S_{b_{ij}}^{{\mathrm{\nu ̃}}_{ijkl}^{\prime \prime }}$$

78 $$S_{a_{ij}}^{{\mathrm{\mu ̃}}_{ijkl}^{\prime \prime }}=X_{{\mathrm{\mu ̃}}_{ijkl}{\mathrm{\mu ̃}}_{ijkl}^{\prime \prime }}^{\mathrm{canon}}S_{{\mathrm{\nu ̃}}_{ij}}^{{\mathrm{\mu ̃}}_{ijkl}}X_{{\mathrm{\nu ̃}}_{ij}a_{ij}}^{\mathrm{PNO}}$$

The corresponding eigenvectors of **D**_**ijkl**_ are called quadruples natural orbitals (QNOs), labeled |*a*_*ijkl*_⟩, and they are truncated using the *T*_CUT_QNO_ cutoff for occupation number. From practical experience, relatively loose cutoffs are required for QNOs (as seen in Table 4) compared to PNOs or TNOs since much of the CI quadruples is accounted for at lower levels of coupled-cluster theory through the $T_{2}^{2}$ , *T*_3_ *T*_1_, $T_{2}T_{1}^{2}$ , and $T_{1}^{4}$ contributions. From our prior work,^109^ we have determined that a relatively loose quadruples natural orbital (QNO) cutoff of 10^–6^ or 3.33 × 10^–7^ is required to accurately compute the (Q) contribution. After experimentation, we found that DLPNO–CCSDTQ is less sensitive than DLPNO–CCSDT­(Q) to the QNO cutoff. Unlike the DLPNO-(Q) energy, the DLPNO–CCSDTQ energy is computed as a function of the singles and doubles amplitude and not directly on the quadruples amplitude. Thus, the quadruples contribution in DLPNO–CCSDTQ is only *indirectly* dependent on the quadruples amplitude. From our testing, we determined that a cutoff of 3.33 × 10^–6^ is sufficient. As later shown in our applications, the Q-(Q) difference is still accurately captured on the order of 0.01 kcal mol^–1^ even with the difference in cutoffs used with the two levels of theory. The full set of all virtual orbitals utilized, showing the flow of information transfer, as well as relevant cutoffs used at each level of theory in the DLPNO–CCSDTQ pipeline, is shown in Figure 2.

#### 3.1.5 Extended Pair Natural Orbitals (XPNOs)

The most expensive term in a DLPNO–CCSDTQ computation is the $T_{mnkl}^{abcd}\to T_{ijkl}^{abcd}$ contribution. In the basis of QNOs, this takes the form ( $G_{ij}^{mn}$ term from eq 50):

79 $$R_{ijkl}^{a_{ijkl}b_{ijkl}c_{ijkl}d_{ijkl}}+=\frac{1}{4}G_{ij}^{mn}T_{mnkl}^{a_{mnkl}b_{mnkl}c_{mnkl}d_{mnkl}}S_{a_{mnkl}b_{mnkl}c_{mnkl}d_{mnkl}}^{a_{ijkl}b_{ijkl}c_{ijkl}d_{ijkl}}$$

where the QNO overlaps are expressed as

80 $$S_{a_{mnkl}b_{mnkl}c_{mnkl}d_{mnkl}}^{a_{ijkl}b_{ijkl}c_{ijkl}d_{ijkl}}=S_{a_{mnkl}}^{a_{ijkl}}S_{b_{mnkl}}^{b_{ijkl}}S_{c_{mnkl}}^{c_{ijkl}}S_{d_{mnkl}}^{d_{ijkl}}$$

Due to the complexity of writing out eq 79, we have devised a more compact notation:

81 $$R_{ijkl}^{abcd}+=\frac{1}{4}G_{ij}^{mn}T_{mnkl}^{abcd}\left(\left|a_{mnkl}\right\rangle \to \left|a_{ijkl}\right\rangle \right)$$

where term $T_{mnkl}^{abcd}\left(\left|a_{mnkl}\right\rangle \to \left|a_{ijkl}\right\rangle \right)$ is shorthand for $T_{mnkl}^{a_{mnkl}b_{mnkl}c_{mnkl}d_{mnkl}}S_{a_{mnkl}b_{mnkl}c_{mnkl}d_{mnkl}}^{a_{ijkl}b_{ijkl}c_{ijkl}d_{ijkl}}$ (i.e., projecting all of its virtual indices from the QNO space of *mnkl* to the QNO space of *ijkl*). For amplitudes-like quantities, their virtual indices are in their corresponding local virtual orbital space—e.g., $R_{ijkl}^{abcd}\equiv R_{ijkl}^{a_{ijkl}b_{ijkl}c_{ijkl}d_{ijkl}}$ .

The cost of projecting the $T_{mnkl}^{abcd}$ amplitudes from the QNO space of $T_{ijkl}^{abcd}$ is $O\left(n_{ijkl}\cdot n_{lmo,ijkl}^{2}\cdot n_{qno,ijkl}^{4}\cdot n_{qno,mnkl}\right)$ , since it effectively requires a loop over all quadruples *ijkl* ( $O\left(n^{4}\right)$ without sparsity), and then an inner nested loop over all *mnkl* that interact with *ijkl* ( $O\left(n^{2}\right)$ worst case), and finally four matrix multiplies over all the virtual indices to fully transform the indices from the QNO space of *mnkl* to the QNO space of *ijkl* ( $O\left(n^{5}\right)$ worst-case). This amounts to a worst-case runtime of $O\left(n^{11}\right)$ , more expensive than the $O\left(n^{10}\right)$ scaling of canonical CCSDTQ. Though the observed scaling of this term is much lower than $O\left(n^{11}\right)$ with the local approximations, it remains a bottleneck in its current form.

To address this bottleneck, we have introduced an additional virtual space based on combined pair natural orbital (PNO) spaces, known as extended pair natural orbitals (or XPNOs). XPNOs for an LMO pair *kl* are defined as eigenvectors of an “extended pair density”:

82 $$D_{\mathrm{kl}}^{\mathrm{ext}}=\frac{1}{N_{mn}}\underset{mn}{\sum }D_{mnkl}=\frac{1}{6N_{nm}}\underset{mn}{\sum }\left(D_{\mathrm{mn}}+D_{\mathrm{mk}}+D_{\mathrm{ml}}+D_{\mathrm{nk}}+D_{\mathrm{nl}}+D_{\mathrm{kl}}\right)$$

This approach is reminiscent of the mathematical relation between local natural orbitals (LNOs) and pair natural orbitals (PNOs), where the LNO density matrix can be computed through a partial sum over a pair density matrix **D**_*i*_ = ∑_*j*_ **D**_*ij*_.^90^ Similar to QNOs, the relevant pair densities are computed through DLPNO–CCSDT doubles amplitudes, and *N*_*mn*_ represents the number of elements *mn* such that *mn*, *mk*, *ml*, *nk*, *nl*, and *kl* all form valid LMO pairs. The resulting natural orbitals that are formed are called extended pair natural orbitals (XPNOs) and are labeled $\left|\overset{\sim }{a_{kl}\;}\right\rangle$ . Using XPNOs, a much more efficient algorithm for the $T_{mnkl}^{abcd}$ contribution can be devised. In the new algorithm involving XPNOs, $T_{mnkl}^{abcd}$ amplitudes from the QNO space of *mnkl* are first projected to the XPNO space of *kl*.

83 $${\mathrm{T̃}}_{mnkl}^{abcd}\left(\left|\overset{\sim }{a_{kl}\;}\right\rangle \right)=T_{mnkl}^{abcd}\left(\left|a_{mnkl}\right\rangle \to \left|\overset{\sim }{a_{kl}\;}\right\rangle \right)$$

The formation of this intermediate has a worst-case $O\left(n^{9}\right)$ complexity, as opposed to $O\left(n^{11}\right)$ for directly projecting |*a_mnkl_* ⟩ → |*a*_*ijkl*_⟩, since the additional $O\left(n^{2}\right)$ loop accounting for interacting *ijkl* over all *mnkl* is not required. After the formation of the projected amplitude, we form another intermediate,

84 $$X_{ijkl}^{abcd}\left(\left|\overset{\sim }{a_{kl}\;}\right\rangle \right)=\frac{1}{4}G_{ij}^{mn}{\mathrm{T̃}}_{mnkl}^{abcd}\left(\left|\overset{\sim }{a_{kl}\;}\right\rangle \right)$$

This worst-case (no sparsity) complexity of this term is the same $O\left(n^{10}\right)$ cost as in canonical CCSDTQ implementations. Finally, the contribution of to $R_{ijkl}^{abcd}$ can be expressed as

85 $$R_{ijkl}^{abcd}+=X_{ijkl}^{abcd}\left(\left|\overset{\sim }{a_{kl}\;}\right\rangle \to \left|a_{ijkl}\right\rangle \right)$$

with the cost of projecting $X_{ijkl}^{abcd}$ from the XPNO space of *kl* to the QNO space of *ijkl* being $O\left(n^{9}\right)$ . The XPNO spaces are designed to be robust, and the “double projection” $\left|a_{mnkl}\right\rangle \to \left|\overset{\sim }{a_{kl}\;}\right\rangle \to \left|a_{ijkl}\right\rangle$ does not incur a significant loss of information. Instead of focusing on summing over *mn* in a minimal occupied coupling space (*mnkl*), the extended pair space of *kl* covers both the extension from occupied *kl* pairs to *mnkl* quadruples and the subsequent coupling of other occupied pairs to the *mnkl* quadruples. As the formation of *mnkl* quadruples with other *ijkl* coupling spaces is driven primarily by overlap, which is transitive, even a loose threshold on extended pair spaces still allows for good coverage of this combined space. Finally, since the coupling space is now “shared” by all *mnkl* quadruples with common *kl*, overlap between *mn* summands for different *kl* can be leveraged. After testing, we found that an XPNO occupation number cutoff of 10^–5^ suffices.

### 3.2 Working Equations

From the working equations given in Section IIB, equations in the LMO/QNO basis can be derived relatively straightforwardly. We first define the formation of the density-fitted two-electron integrals in the localized occupied, virtual, and auxiliary fitting domains of *ijkl*:

86 $$B_{mi}^{Q}\left[ijkl\right]≡B_{m_{ijkl}i}^{Q_{ijkl}}={\left(Q_{ijkl}|P_{ijkl}\right)}^{-\frac{1}{2}}\left(P_{ijkl}|m_{ijkl}i\right)$$

87 $$B_{ai}^{Q}\left[ijkl\right]≡B_{a_{ijkl}i}^{Q_{ijkl}}={\left(Q_{ijkl}|P_{ijkl}\right)}^{-\frac{1}{2}}\left(P_{ijkl}|{\mathrm{\mu ̃}}_{ijkl}i\right)X_{{\mathrm{\mu ̃}}_{ijkl}a_{ijkl}}^{\mathrm{QNO}}$$

88 $$B_{ma}^{Q}\left[ijkl\right]≡B_{m_{ijkl}a_{ijkl}}^{Q_{ijkl}}={\left(Q_{ijkl}|P_{ijkl}\right)}^{-\frac{1}{2}}\left(P_{ijkl}|m{\mathrm{\mu ̃}}_{ijkl}\right)X_{{\mathrm{\mu ̃}}_{ijkl}a_{ijkl}}^{\mathrm{QNO}}$$

89 $$B_{ab}^{Q}\left[ijkl\right]≡B_{a_{ijkl}b_{ijkl}}^{Q_{ijkl}}={\left(Q_{ijkl}|P_{ijkl}\right)}^{-\frac{1}{2}}\left(P_{ijkl}|{\mathrm{\mu ̃}}_{ijkl}{\mathrm{\nu ̃}}_{ijkl}\right)X_{{\mathrm{\mu ̃}}_{ijkl}a_{ijkl}}^{\mathrm{QNO}}X_{{\mathrm{\nu ̃}}_{ijkl}b_{ijkl}}^{\mathrm{QNO}}$$

These integrals can be used to form the necessary *t*_1_-dressed integrals and Fock matrix elements, with the necessary *t*_1_ amplitudes being projected from their diagonal PNO space to the QNO space of *ijkl*. For example,

90 $${\mathrm{B̃}}_{ab}^{Q}\left[ijkl\right]=B_{ab}^{Q}\left[ijkl\right]-t_{k}^{a}\left(\left|a_{kk}\right\rangle \to \left|a_{ijkl}\right\rangle \right)\cdot B_{kb}^{Q}\left[ijkl\right]$$

For reader comprehension, an example from the Fock matrices is also given

91 $${\mathrm{F̅}}_{mi}\left[ijkl\right]=F_{mi}\left[ijkl\right]+\left(2B_{mi}^{Q}B_{ne}^{Q}-B_{me}^{Q}B_{ni}^{Q}\right)\left[ijkl\right]\cdot t_{n}^{e}\left(\left|a_{nn}\right\rangle \to \left|a_{ijkl}\right\rangle \right)$$

92 $${\mathrm{F̃}}_{mi}\left[ijkl\right]={\mathrm{F̅}}_{mi}\left[ijkl\right]+{\mathrm{F̅}}_{ma}\left[ijkl\right]\cdot t_{i}^{a}\left(\left|a_{ii}\right\rangle \to \left|a_{ijkl}\right\rangle \right)$$

For the quadruple contribution to the doubles residual, by exploiting the symmetry of doubles amplitudes,

93 $$R_{ij}^{ab}+=\underset{m,n\in \left\{m_{ij},n_{ij}\right\}}{\sum }\frac{1}{2}\left[\left(B_{me}^{Q}B_{nf}^{Q}\right)\left[mnij\right]\cdot \beta_{mnij}^{efab}\right]\left(\left|a_{mnij}\right\rangle \to \left|a_{ij}\right\rangle \right)$$

The parentheses denote the order of operations, with the integrals in the QNO space of *ijkl* being contracted with the β amplitude first, before the virtual space resulting in the expression being projected from the QNO space of *ijkl* to the PNO space of *kl*. This term is far from a bottleneck, scaling $O\left(n^{8}\right)$ worst case, or $O\left(n_{ijkl}\cdot n_{qno,ijkl}^{4}\right)$ in terms of domain sizes, the same cost as indexing over all the elements in a quadruples amplitude, and typically does not contribute much to the overall runtime of a DLPNO–CCSDTQ computation. For the triples residual, the three contributions are

94 $$R_{ijk}^{abc}+=\left[{\mathrm{F̃}}_{me_{ijk}}S_{e_{ijk}}^{e_{mijk}}\cdot \alpha_{mijk}^{eabc}\right]\left(\left|a_{mijk}\right\rangle \to \left|a_{ijk}\right\rangle \right)$$

95 $$R_{ijk}^{abc}+=P_{ia/jb,kc}\left(\left[S_{e_{ijk}}^{e_{mijk}}\left({\mathrm{B̃}}_{ae}^{Q}B_{mf}^{Q}\right)S_{f_{ijk}}^{f_{mijk}}\cdot \alpha_{mijk}^{febc}\right]\left(\left|a_{mijk}\right\rangle \to \left|a_{ijk}\right\rangle \right)\right)$$

96 $$R_{ijk}^{abc}-=P_{ia/jb,kc}\left(\left[S_{e_{ijk}}^{e_{mnjk}}\left(B_{me}^{Q}{\mathrm{B̃}}_{ni}^{Q}\right)\cdot \alpha_{mnjk}^{eabc}\right]\left(\left|a_{mnjk}\right\rangle \to \left|a_{ijk}\right\rangle \right)\right)$$

where $P_{ia/jb,kc}X_{ijk}^{abc}=X_{ijk}^{abc}+X_{jik}^{bac}+X_{kji}^{cba}$ . In these triples residual equations, all the integrals (two-electron integrals and Fock matrix elements) are first computed in the corresponding triples space *ijk*, before being projected to the corresponding quadruples natural orbital (QNO) space—i.e., in eq 94, the Fock matrix is computed in the TNO space of *ijk* before being projected to the QNO space of *mijk*. In the final step, the resulting quantity is projected into the TNO space of *ijk*. This is mathematically more efficient since it avoids the explicit projection of quadruples amplitudes and involves the projection of, at most, a six-index quantity. For example, the projection from $\alpha_{mnjk}^{eabc}$ to the TNO space of *ijk* in eq 96 would be $O\left(n^{10}\right)$ worst-case.

For the quadruples residual $R_{ijkl}^{abcd}$ , the expressions can be derived using analogous rules that are used in the formation of the doubles and triples residuals—i.e., all integrals and Fock matrix elements are formed directly in the QNO space of *ijkl*, all amplitudes are projected from their relevant spaces (doubles amplitudes are projected from their corresponding PNO space to the QNO space of *ijkl*, and likewise, triples amplitudes are projected from their corresponding TNO space to the QNO space of *ijkl*). There are select terms that were modified for the purposes of avoiding expensive virtual space projections, similar to the $T_{mnkl}^{abcd}$ contribution to $R_{ijkl}^{abcd}$ mentioned previously. For brevity, only the terms that involve expensive projections, in which we made exceptions to the rules, will be presented here, while the full set of working equations will be provided in the Supporting Information. For the contribution of the quadruples amplitude to the $L_{ijk}^{abm}$ intermediate (last term of eq 48), by the traditional application of our rules,

97 $$L_{ijk}^{abm}\left[ijkl\right]+=\frac{1}{2}P_{ij}^{ab}\left[\left(B_{me}^{Q}B_{nf}^{Q}\right)\left[ijkl\right]\cdot \alpha_{nijk}^{fabe}\left(\left|a_{nijk}\right\rangle \to \left|a_{ijkl}\right\rangle \right)\right]$$

This involves an expensive $O\left(n^{10}\right)$ projection from the QNO space of *nijk* to *ijkl*, exacerbated by the fact that this needs to be evaluated over all possible ordered choices of *ijk* out of *ijkl* (24 evaluations). To reduce the cost of this term, the equation is first evaluated in the PNO basis of *ij*, and then projected to the QNO space of *ijkl*,

98 $$\delta L_{ijk}^{abm}\left[ij\right]=\left(B_{me}^{Q}B_{nf}^{Q}\right)\left[ij\right]\cdot \alpha_{nijk}^{fabe}\left(\left|a_{nijk}\right\rangle \to \left|a_{ij}\right\rangle \right)$$

99 $$L_{ijk}^{abm}\left[ijkl\right]+=\delta L_{ijk}^{abm}\left[ij\right]\left(\left|a_{ij}\right\rangle \to \left|a_{ijkl}\right\rangle \right)$$

The cost of this term is now reduced to $O\left(n^{9}\right)$ worst case. The larger size of PNO spaces relative to QNO spaces minimizes the error induced through the projection from |*a*_*nijk*_⟩ → |*a*_*ij*_⟩ → |*a*_*ijkl*_⟩. Another problematic term is the *nmjk* contribution to the $M_{ejk}^{abc}$ intermediate (second term of eq 49),

100 $$M_{ejk}^{abc}\left[ijkl\right]-=\frac{1}{2}P_{jk}^{bc}\left[\left(B_{me}^{Q}B_{nf}^{Q}\right)\left[ijkl\right]\cdot \alpha_{nmjk}^{fabc}\left(\left|a_{nmjk}\right\rangle \to \left|a_{ijkl}\right\rangle \right)\right]$$

This is another term that involves nonfavorable $O\left(n^{11}\right)$ QNO projection operations, as well as a $O\left(n^{11}\right)$ matrix multiplication induced from contracting the integrals with the amplitude. A similar approach is applied here as in the previous case, where everything is first computed in the PNO space of *jk* before being projected onto the QNO space of *ijkl*.

101 $$\delta M_{ejk}^{abc}\left[jk\right]=-\left(B_{me}^{Q}B_{nf}^{Q}\right)\left[jk\right]\cdot \alpha_{nmjk}^{fabc}\left(\left|a_{nmjk}\right\rangle \to \left|a_{jk}\right\rangle \right)$$

102 $$M_{ejk}^{abc}\left[ijkl\right]+=M_{ejk}^{abc}\left[jk\right]\left(\left|a_{jk}\right\rangle \to \left|a_{ijkl}\right\rangle \right)$$

This also reduces the cost of the worst-case $O\left(n^{11}\right)$ operation down to $O\left(n^{9}\right)$ .

### 3.3 Incorporating the Symmetry of the T4 Amplitudes

To minimize the memory demands of storing a complete set of quadruples amplitudes $t_{ijkl}^{abcd}$ , we only store quadruples amplitudes of restricted occupied indices *i* ≤ *j* ≤ *k* ≤ *l*. This is possible due to the column permutation symmetry of quadruples amplitudes:

103 $$t_{ijkl}^{abcd}=t_{jikl}^{bacd}=t_{kjil}^{cbad}=...=t_{lkji}^{dcba}$$

In addition, the $P_{ijkl}^{abcd}$ permutation operator in eq 50 generates 24 terms, which means that some of the expensive tensor contractions may be repeated up to 24 times in a naive implementation. Fortunately, many of these terms possess column permutational symmetry, which can be utilized to reduce the number of contractions that need to be performed. For the two most expensive terms, which are the $T_{mnkl}^{abcd}$ and $T_{ijkl}^{efcd}$ contributions to $R_{ijkl}^{abcd}$ ( $G_{ij}^{mn}$ and $H_{ef}^{ab}$ contributions from eq 50), this symmetry reduces the number of requisite tensor contractions from 24 down to 6:

104 $$R_{ijkl}^{abcd}+=\underset{i\leq j\leq k\leq l}{\sum }G_{ij}^{mn}T_{mnkl}^{abcd}+G_{ik}^{mn}T_{mnjl}^{acbd}+G_{il}^{mn}T_{mnjk}^{adbc}+G_{jk}^{mn}T_{mnil}^{bcad}+G_{jl}^{mn}T_{mnik}^{bdac}+G_{kl}^{mn}T_{mnij}^{cdab}$$

105 $$R_{ijkl}^{abcd}+=\underset{i\leq j\leq k\leq l}{\sum }H_{ef}^{ab}T_{ijkl}^{efcd}+H_{ef}^{ac}T_{ikjl}^{efbd}+H_{ef}^{ad}T_{iljk}^{efbc}+H_{ef}^{bc}T_{jkil}^{efad}+H_{ef}^{bd}T_{jlik}^{efac}+H_{ef}^{cd}T_{klij}^{efab}$$

The factor of $\frac{1}{4}$ disappears due to each contraction in the series accounting for four terms each. These expressions can be shortened using our quadruples “permutation operators” over the virtual indices, as defined in prior work on our DLPNO–CCSDT­(Q) algorithm.^109^ To recap, the permutation operator *P*_(*i*,*j*,*k*,*l*)_ as well as its adjoint $P_{\left(i,j,k,l\right)}^{†}$ are defined as, with (*ĩ*, *j̃*, *k̃*, *ĩ*) representing the tuple (*i*, *j*, *k*, *l*) in ascending order:

106 $$P_{\left(i,j,k,l\right)}X\left(a,b,c,d\right)=X\left(v\left[ĩ\right],v\left[\mathrm{j̃}\right],v\left[\mathrm{k̃}\right],v\left[\mathrm{l̃}\right]\right)$$

107 $$P_{\left(i,j,k,l\right)}^{†}X\left(v\left[ĩ\right],v\left[\mathrm{j̃}\right],v\left[\mathrm{k̃}\right],v\left[\mathrm{l̃}\right]\right)=X\left(a,b,c,d\right)$$

The *v* represents an “index mapping”, with

108 v[ĩ]=a,v[j̃]=b,v[k̃]=c,v[l̃]=d

For example, if *k* ≤ *l* ≤ *j* ≤ *i*, then

109 $$P_{\left(i,j,k,l\right)}X\left(a,b,c,d\right)=X\left(c,d,b,a\right)$$

110 $$P_{\left(i,j,k,l\right)}^{†}X\left(c,d,b,a\right)=X\left(a,b,c,d\right)$$

With these operators, the $T_{mnkl}^{abcd}$ and $T_{ijkl}^{efcd}$ terms can be compactly formulated as

111 $$R_{ijkl}^{abcd}+=\underset{\left(\begin{array}{l} ijkl\\ i^{\prime }j^{\prime }/k^{\prime }l^{\prime } \end{array}\right)}{\sum }P_{\left(i^{\prime },j^{\prime },k^{\prime },l^{\prime }\right)}\left[G_{i^{\prime }j^{\prime }}^{mn}T_{mnk^{\prime }l^{\prime }}^{abcd}\right]$$

112 $$R_{ijkl}^{abcd}+=\underset{\left(\begin{array}{l} ijkl\\ i^{\prime }j^{\prime }/k^{\prime }l^{\prime } \end{array}\right)}{\sum }P_{\left(i^{\prime },j^{\prime },k^{\prime },l^{\prime }\right)}\left[H_{ef}^{ab}T_{i^{\prime }j^{\prime }k^{\prime }l^{\prime }}^{efcd}\right]$$

where the notation $\left(\begin{array}{l} ijkl\\ i^{\prime }j^{\prime }/k^{\prime }l^{\prime } \end{array}\right)$ represents (*i*′, *j*′) being selected from the pair list

{(*i*, *j*), (*i*, *k*), (*i*, *l*), (*j*, *k*), (*j*, *l*), (*k*, *l*)} and (*k*′, *l*′) representing the complement—e.g., if (*i*′, *j*′) = (*j*, *l*), then (*k*′, *l*′) = (*i*, *k*). Similar expressions exist for other terms in eq 50 and will be provided in the Supporting Information.

### 3.4 Convergence Acceleration

Due to the large number of independent parameters involved in an iterative CCSDTQ procedure, most CCSDTQ codes are notoriously difficult to converge, especially for systems with significant multireference character. To mitigate this issue, our algorithm uses a combination of DIIS (direct inversion of the iterative subspace),^148,149^ and CCSD microiterations to assist with convergence.^150,151^ With the CCSD microiterations, our algorithm uses a simplified version of the algorithm introduced by Matthews and Stanton,^150^ running by default, three *T*_2_ and *T*_1_ updates every DLPNO–CCSDTQ iteration, to allow for triples and quadruples effects to “saturate” in the energy expressions. The number of microiterations can be set by the user through the “DLPNO_QUADS_MICROITERATIONS” keyword. DIIS is performed every “full” iteration as well in order to also handle the multireference systems that exhibit poor convergence behavior. By default, DIIS extrapolation is used for all amplitudes but can be turned off for the *T*_4_ amplitudes on larger systems where memory limitations are encountered, by setting the “EXTRAPOLATE_T4” keyword to false. In addition, the pseudoinverse approach of Matthews and Stanton is also utilized for the triples and quadruples amplitudes during each iteration to remove linear dependencies, the formulas for these can be found in eqs 27 and 28 in ref 52. Through a combination of these techniques, as well as a significantly reduced number of independent parameters that need to be optimized in DLPNO–CCSDTQ compared to canonical CCSDTQ ( O(n) vs $O\left(n^{8}\right)$ ), we have not found convergence difficulties in any of the systems that are studied in this work, including the multireference CH_2_OO Criegee intermediate, as well as ClF_3_.

## 4 Implementation Details

The frozen-core approximation is used for all correlated computations, and density-fitting is not utilized for the preceding Hartree–Fock computation. To verify the accuracy of our derivations, we prototyped a canonical density-fitted CCSDTQ implementation in Python with the Psi4Numpy interface^152^ and compared the results to the implementation in CFOUR with an exact auxiliary basis set using function products.^52,153^ Once our code was written in C++, it was tested with cc-pVXZ-RI auxiliary basis sets and all local truncations set to zero against the Psi4Numpy implementation. Our code is currently available in a developmental branch of the free and open-source Psi4 package on GitHub (version 1.10).^154^ For all tensor contractions and transpositions, the Einsums library is used (version 1.0.6), which allows most of the tensor operations in this work to be written in generalized Einstein summation notation, while taking advantage of efficient GEMM/GEMV subroutines for matrix multiplication or matrix-vector products.^155^ The necessary low-level BLAS calls like GEMM/GEMV are determined at compile time from the written Einstein summation expressions. The algorithm is also parallelized with OpenMP over all quadruplets *ijkl*. Each quadruplet is sorted by the size of its QNO space and assigned to each thread in descending order to maximize the parallel efficiency of the algorithm on multicore nodes. From our tests, we find that 70–80% CPU efficiency is achieved with 32 CPU cores, which we deem more than acceptable considering that all of the density-fitted two-electron integrals are written to disk. Many of the small tensors associated with each quadruplet *ijkl* are computed in core over each thread, so the memory cost scales linearly with the number of CPU cores, though for larger systems, it is never more than the cost required to store the quadruples amplitudes.

Unless otherwise stated, all parameters used in this paper are evaluated with the values given in Table 1 for LMO-based parameters, Table 2 for local RI parameters, Table 3 for PAO-based parameters, and Table 4 for PNO/TNO/QNO/XPNO-based parameters. Unless otherwise stated, all computations are run using AMD EPYC 9274F (Genoa) CPUs, with a base clock of 4.05 GHz and a max clock of 4.3 GHz. For timings, 48 CPU cores and up to 2880 GB of RAM were utilized. All expensive ERIs (e.g., (*Q*| *ia*) and (*Q*| *ab*) type integrals in the PNO, TNO, and QNO basis) are written to disk, as well as the error vectors utilized in DIIS extrapolation. All other quantities are kept in RAM, including the $t_{ijkl}^{abcd}$ amplitudes.

**Table 1:** Default Values for LMO-Related Parameters in DLPNO–CCSDTQ

Parameter	Description	Value
*T*_CUT_PRE_	Energy cutoff for determining dipole pairs	**10** ^**–10**^
*T*_CUT_DO_IJ_	Overlap cutoff used in the determination of “dipole pairs”	**10** ^**–5**^
*T*_CUT_PAIRS_MP2_	Energy cutoff for determining semicanonical MP2 pairs	**10** ^**–9**^
*T*_CUT_PAIRS_	Energy cutoff for determining strong and weak pairs	**10** ^**–8**^
*T*_CUT_TRIPLES_WEAK_	Energy cutoff for prescreening triplets	**10** ^**–8**^
*T*_CUT_QUADS_WEAK_	Energy cutoff for prescreening quadruplets	**10** ^**–8**^
TRIPLES_MAX_WEAK_PAIRS	Max number of weak pairs *ij*, *jk*, *ik* in initial triplets *ijk*	**2**
QUADS_MAX_WEAK_PAIRS	Max number of weak pairs in initial quadruplets *ijkl*	**3**

**Table 2:** Default Values for Local DF/RI-Related Parameters in DLPNO–CCSDTQ

Parameter	Description	Value
*T*_CUT_MKN_	Mulliken local RI function tolerance for pairs (*P*_*ij*_, *Q*_*ij*_)	**10** ^**–4**^
*T*_CUT_MKN_TRIPLES_PRE_	Mulliken local RI function tolerance in DLPNO-(T_0_) prescreening	**10** ^**–1**^
*T*_CUT_MKN_TRIPLES_	Mulliken local RI function tolerance for triplets (*P*_*ijk*_, *Q*_*ijk*_)	**10** ^**–2**^
*T*_CUT_MKN_QUADS_PRE_	Mulliken local RI function tolerance in DLPNO-(Q_0_) prescreening	**10** ^**–1**^
*T*_CUT_MKN_QUADS_	Mulliken local RI function tolerance for quadruplets (*P*_*ijkl*_, *Q*_*ijkl*_)	**10** ^**–2**^

**Table 3:** Default Values for the Tolerance in Assigning PAO-Related Parameters in DLPNO–CCSDTQ

Parameter	Description	Value
*T*_CUT_DO_	To pair domains (*μ̃*_*ij*_, *ν̃*_*ij*_)	**5** × **10** ^**–3**^
*T*_CUT_DO_TRIPLES_PRE_	In DLPNO-(T_0_) prescreening	**2** × **10** ^**–2**^
*T*_CUT_DO_TRIPLES_	To triplet domains (*μ̃*_*ijk*_, *ν̃*_*ijk*_)	**10** ^**–2**^
*T*_CUT_DO_QUADS_PRE_	In DLPNO-(Q_0_) prescreening	**2** × **10** ^**–2**^
*T*_CUT_DO_QUADS_	To triplet domains (*μ̃*_*ijk*_, *ν̃*_*ijk*_)	**10** ^**–2**^

**Table 4:** Default Values for All Natural Orbital Related Parameters in DLPNO–CCSDTQ^a^

Parameter	Description	Value
*T*_CUT_PNO_MP2_	PNO occupation cutoff used in DLPNO-MP2 computation	**10** ^**–10**^
*T*_CUT_TRACE_MP2_	PNO trace cutoff used in DLPNO-MP2 computation	**0.9999**
*T*_CUT_ENERGY_MP2_	PNO energy cutoff used in DLPNO-MP2 computation	**0.999**
*T*_CUT_PNO_	PNO occupation cutoff used in DLPNO–CCSD computation	**10** ^**–8**^
*T*_CUT_TRACE_	PNO trace cutoff used in DLPNO-MP2 computation	**0.999**
*T*_CUT_ENERGY_	PNO energy cutoff used in DLPNO-MP2 computation	**0.997**
*T*_CUT_TNO_	TNO occupation cutoff used in DLPNO–CCSD(T) computation	**10** ^**–9**^
*T*_CUT_TNO_FULL_	TNO occupation cutoff used in DLPNO–CCSDT computation	**10** ^**–7**^
*T*_CUT_QNO_	QNO occupation cutoff used in DLPNO-(Q) computation	**10** ^**–6**^
**T**_CUT_QNO_FULL_	QNO occupation cutoff used in DLPNO–CCSDTQ computation	**3.33** × **10** ^**–6**^
**T**_CUT_XPNO_	XPNO occupation cutoff used in DLPNO–CCSDTQ computation	**10** ^**–5**^

a All energy related cutoffs are given in Hartrees (*E_h_*). The last two parameters are introduced in this work and **bolded.**

## 5 Results

### 5.1 Convergence Relative to Canonical CCSDTQ

To verify the convergence of our DLPNO–CCSDTQ algorithm with respect to canonical CCSDTQ, we compute DLPNO–CCSDTQ correlation energies at a series of QNO cutoffs for HCN, using an AE-CCSDTQ­(P)/CBS + MVD1 geometry and canonical CCSDTQ energies derived from the work of Allen et al.^115^ For these computations, all natural orbital tolerances have been set to zero, with the exception of the QNO tolerance (Table 4). In Figure 3, we plot the convergence of DLPNO–CCSDTQ/cc-pVDZ correlation energies at each QNO cutoff relative to canonical CCSDTQ/cc-pVDZ. To minimize density-fitting error, the cc-pV5Z-RI auxiliary basis was utilized for ERIs used in correlated computations. From these tests, we find a QNO cutoff value of 3.33 × 10^–6^ acceptable for the purposes of accuracy, as it yields absolute energy errors within 8 μEh (or 0.005 kcal mol^–1^), satisfying our target accuracy range of 0.01–0.05 kcal mol^–1^ for relative energies in applications of our method. At a QNO cutoff of 0.0, we determined that the error (around 0.6 μEh) can be completely attributed to density-fitting through comparing DLPNO-MP2 results to canonical MP2 results.

**Figure 3:**
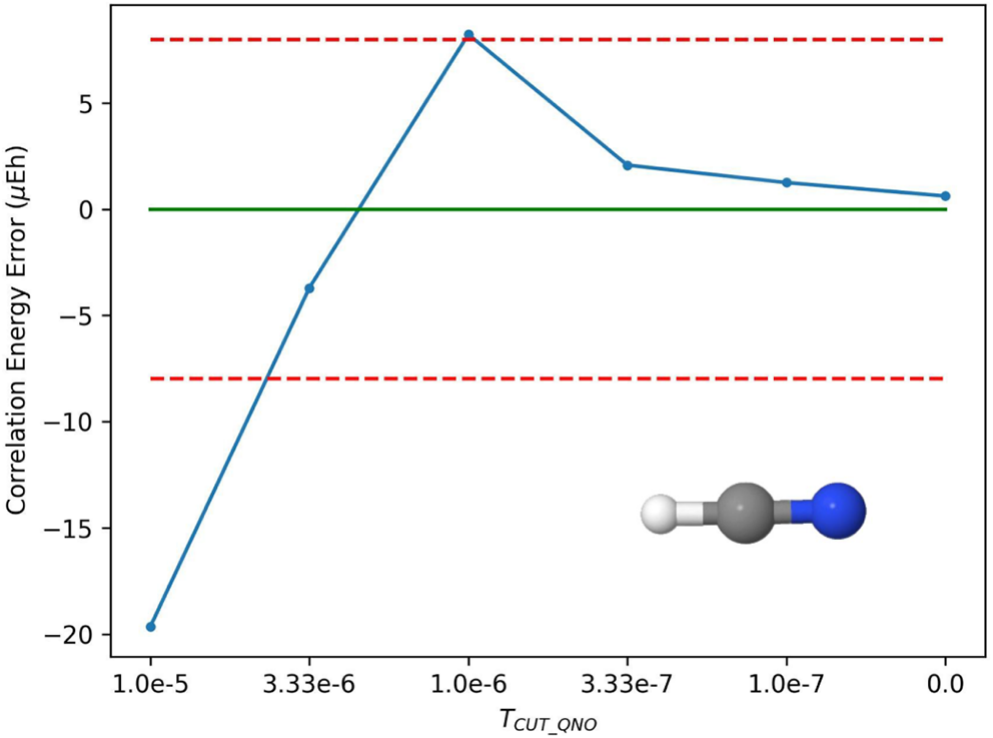
The difference between total DLPNO–CCSDTQ correlation energy and total canonical CCSDTQ correlation energies with respect to QNO tolerance. QNO cutoff is the only independent variable, as all other thresholds have been set to zero. The dashed red lines indicate the target accuracy range of ±8 μEh (or 0.005 kcal mol^–1^), for intended applications of DLPNO–CCSDTQ.

### 5.2 Reaction Energies

#### 5.2.1 HCNO Reaction

Next, we tested our DLPNO–CCSDTQ method on the HCN + O (^3^ *P*) → HCNO reaction, also from the prior work of Allen et al.^115^ from which geometries and canonical CCSDTQ energies from all species are available. The geometries are optimized at the AE-CCSDTQ­(P)/CBS + MVD1 level, and canonical CCSDTQ energies are available at the cc-pVDZ level of theory. For our computations, the open-shell UHF CCSDTQ energy for O (^3^ *P*) is taken from the work of Allen et al., while our DLPNO–CCSDTQ algorithm is used for the closed-shell HCN and HCNO species. The results are presented in Table 5, and they show that taking the difference between DLPNO–CCSDTQ and DLPNO–CCSDT­(Q) energies exactly matches the difference between canonical CCSDTQ and CCSDT­(Q), in spite of larger errors (on the order of 0.1 kcal mol^–1^) occurring at lower levels of theory. The overall post-CCSD­(T) difference computed by using DLPNO–CCSDTQ and DLPNO–CCSD­(T) differences also shows a difference of only 0.01 kcal mol^–1^. This would make a composite scheme such as [DLPNO–CCSDTQ – DLPNO–CCSD­(T)] + CCSD­(T) a viable method for larger molecules in which ultra-accuracy is required, since most of the local correlation errors are accounted for at the CCSD­(T) level or lower.

**Table 5:** Reaction Energies for HCN + O (^3^ *P*) → HCNO (kcal mol^–1^) at the cc-pVDZ Level of Theory^a^

Method	Canonical (Ref. 115)	DLPNO (This work)
HF	+19.77	+19.77
δMP2	–65.98	–66.02
δCCSD	+16.12	+16.01
δCCSD(T)	–5.92	–5.93
δCCSDT	+0.22	+0.22
δCCSDT(Q)	–0.96	–0.95
δCCSDTQ	0.24	0.24
**Total**	–36.50	–36.65
**Total post-CCSD(T)**	–0.50	–0.49

a The energy difference in each row with a *δ* denotes the reaction energy computed at the given level of theory subtracted from the prior reaction (e.g., *δ*CCSDTQ ≡ CCSDTQ – CCSDT­(Q) for canonical and *δ*CCSDTQ ≡ DLPNO–CCSDTQ – DLPNO–CCSDT­(Q) in the DLPNO column).

#### 5.2.2 Criegee Intermediates

To further evaluate this paradigm, we also computed reaction energies for the CH_2_OO enthalpy of formation, by combining the enthalpy of formation for HCOOH from the active thermochemical tables (ATcT),^156^ as well as the energy difference of HCOOH and CH_2_OO.^114^ Criegee intermediates such as CH_2_OO are a good test case for CCSDTQ due to their multireference character. Our geometries are derived from the work of Begley et al., and we also compare our results to the optimized virtual orbital (OVO) approach^87^ (with a 75% virtual space) that they presented, given in Table 6. Once again, our algorithm exactly captures the CCSDTQ-CCSDT­(Q) difference within a resolution of 0.01 kcal mol^–1^, and the results show that much of the local correlation error is accounted for at the CCSD­(T) level and lower. In addition, our DLPNO hierarchy of methods is much more reliable at capturing post-CCSD­(T) effects than the OVO approach.

**Table 6:** Comparison of DLPNO, OVO, and Canonical Results for the CH_2_OO Enthalpy of Formation (kcal mol^–1^) at the cc-pVDZ Level of Theory, the Energy Difference in Each Row with a *δ* Denotes the Reaction Energy Computed at the Given Level of Theory Subtracted from the Prior (e.g., *δ*CCSDTQ ≡ CCSDTQ – CCSDT­(Q) for Canonical, *δ*CCSDTQ ≡ OVO–CCSDTQ – OVO–CCSDT­(Q) for OVO, and *δ*CCSDTQ ≡ DLPNO–CCSDTQ – DLPNO–CCSDT­(Q) in the DLPNO Column)

Method	Canonical (Ref. 114)	OVO (Ref. 114)	DLPNO (This work)
HF	+47.75	+47.75	+47.78
δMP2	–6.53	–7.11	–6.48
δCCSD	–6.48	–6.91	–6.78
δCCSD(T)	–4.76	–4.77	–4.72
δCCSDT	–0.19	–0.22	–0.17
δCCSDT(Q)	–1.39	–1.19	–1.38
δCCSDTQ	0.68	0.57	0.68
**Total**	28.63	28.04	28.93
**Total post-CCSD(T)**	–0.90	–0.84	–0.87

#### 5.2.3 ClF_3_ Formation

For comparisons of our code against larger canonical CCSDTQ computations, we decided to compute reaction energies involving the formation of the multireference ClF_3_ molecule from the W4–17 data set,^157^ with the cc-pVDZ and aug-cc-pVDZ basis sets—the geometry given in Figure 4. In Table 7, we present results for energy differences as well as focal point increments as well as number of quadruples natural orbitals (QNOs) retained. Canonical CCSDT, CCSDT­(Q), and CCSDTQ results were obtained using CFOUR.^52,153^ All the relevant geometries (ClF_3_, ClF, and F_2_) can be found in the Supporting Information of ref 157. All computed absolute energies are given in the Supporting Information SI. These results suggest that our chosen cutoffs indeed capture the quadruple effects, with the error in the focal point increments being around 0.02 to 0.03 kcal mol^–1^ in the cc-pVDZ case and 0.01 kcal mol^–1^ in the aug-cc-pVDZ case. The lower error with the larger basis set is likely due to more fortuitous error cancellation when larger basis sets are used (i.e., the error caused by PNO truncation is proportionately smaller for ClF and F2 compared to ClF3 in cc-pVDZ, as opposed to aug-cc-pVDZ), evidenced by the large discrepancy in the CCSDT energies at the cc-pVDZ level (around 0.23 kcal mol^–1^), as opposed to aug-cc-pVDZ (0.03 kcal mol^–1^). Overall, this is very encouraging, since for more rigorous studies on larger systems, the amount of error cancellation is expected to be similar. In addition, the average number of QNOs retained is very similar across both reactants and products (13–15 for cc-pVDZ and 20–21 for aug-cc-pVDZ), suggesting that our chosen QNO cutoff of 3.33 × 10^–6^ is sufficiently converged and would be applicable for larger systems.

**Figure 4:**
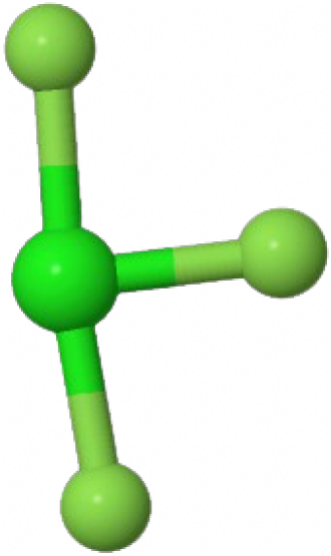
Geometry of ClF_3_ used in this study, from W4–17.^157^

**Table 7:** Reaction Energy for ClF_3_ Formation from ClF and F_2_ (kcal mol^–1^) at the cc-pVDZ (DZ) and aug-cc-pVDZ (aDZ) Basis Sets with DLPNO–CCSDTQ and Canonical CCSDTQ^a^

	CCSDT	CCSDT(Q)	CCSDTQ	δQ-T	δQ-(Q)	*n*_virt,ijkl_ (ClF_3_)	*n*_virt,ijkl_ (ClF)	*n*_virt,ijkl_ (F_2_)
Canonical/DZ	6.71	6.62	6.69	–0.023	+0.070	38 (38–38)	19 (19–19)	19 (19–19)
DLPNO/DZ	6.48	6.37	6.47	–0.003	+0.102	15 (8–23)	14 (9–19)	13 (9–18)
Canonical/aDZ	–13.83	–13.85	–13.78	+0.054	+0.070	74 (74–74)	37 (37–37)	37 (37–37)
DLPNO/aDZ	–13.80	–13.82	–13.75	+0.047	+0.063	20 (12–29)	21 (15–28)	20 (15–27)

a δQ-T denotes a CCSDTQ-CCSDT energy difference, and δQ-(Q) denotes CCSDTQ-CCSDT­(Q). *n*_virt,ijkl_ denotes number of virtual orbitals used in the quadruples space, or number of canonical virtual orbitals in non-DLPNO case. The parentheses denote a mininum to maximum range.

### 5.3 Large Systems

#### 5.3.1 Benzene Dimer (π–π)

To highlight the capabilities of our code, we performed a DLPNO–CCSDTQ computation on a benzene dimer (π–π sandwich configuration) in the cc-pVDZ basis set (30 active occupied orbitals and 186 virtual orbitals). The geometry features an interplanar distance of 3.9 Å and is taken from the S22A test set in ref 158, and is visually depicted in Figure 5. Counterpoise (CP) correction is used in the monomer computations. For the dimer computation, each DLPNO–CCSDTQ iteration took around 3 h, and the max RAM utilization was 926 GB. For the monomer, each DLPNO–CCSDTQ iteration took around 45 min, and up to 286 GB of RAM was utilized. For the dimer, an average size of the quadruple virtual space was 23 QNOs out of 186 possible, while 9346 quadruplets survived the initial DLPNO-(Q_0_) prescreening step out of 40020 possible. Based on this, we estimate the computation on the benzene dimer to take around 400 years per iteration for canonical CCSDTQ. This is estimated by scaling our time by the most expensive step of a canonical CCSDTQ computation $\left[O\left(o^{4}v^{6}\right)\right]$ , with $\left(3\mathrm{hours}\times {\left(\frac{186}{23}\right)}^{6}\times \left(\frac{40020}{9346}\right)\sim 400\mathrm{years}\right)$ . Even with more advanced vectorization and incorporation of full non-Abelian point group symmetry, the computation would still likely take many years! Assuming a speedup of *h* ^2^ = 12^2^ = 144 for the *D*_6*h*_ point group, and a ballpark estimate of a maximal efficiency speedup of 10× from preliminary tests with the canonical CCSDTQ implementation in CFOUR,^153^ each iteration would still take more than 3 months, and over 5 years total assuming 20 iterations). This would only be the *best-case* scenario, as other configurations of the benzene dimer are not as highly symmetric, and point group symmetry and vectorization do not fundamentally change the $O\left(n^{10}\right)$ scaling, as opposed to our linear-scaling DLPNO–CCSDTQ algorithm.

**Figure 5:**
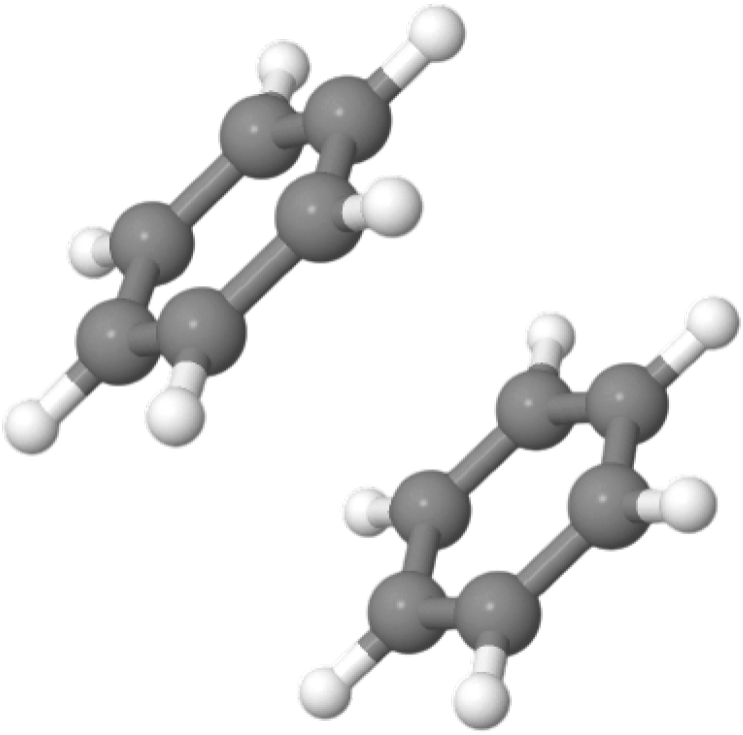
Geometry of the benzene dimer used in this study (ref 158).

We present our interaction energy results in Table 8. The success of CCSD­(T) can be shown in the table, where the impact of full triples and perturbative quadruples almost exactly cancels out. From this preliminary computation, we find that full quadruples may contribute an additional 0.1 kcal mol^–1^ to the interaction energy of a benzene dimer, and this is non-negligible in the context of noncovalent interactions. We note that, however, this is a preliminary result that is meant to showcase an application of our code in future studies and is not designed to be a rigorously benchmarked value. Nonetheless, this is, to our knowledge, the first computation performed using a dimerized conjugated aromatic system using coupled-cluster theory up to full quadruples excitations, though computations up to perturbative quadruples have been performed.^159^ This could have significant implications in the recent controversy between CCSD­(T) and Fixed-Node Diffusion Monte Carlo (FN-DMC) on noncovalent interactions in larger conjugated systems.^160,161^ Once again, we emphasize that this warrants further investigation, as this computation represents only a demonstrative case of an application of our code. Further studies involving parallel-displaced (PD) and T-shaped dimer (TS) configurations, other conjugated π systems, and larger basis sets (per available computing resources) will be of interest.^162,163^

**Table 8:** Intermolecular Interaction Energy for Benzene Dimer (π–π Sandwich Configuration) (kcal mol^–1^) at the cc-pVDZ Level of Theory, the Energy Difference in Each Column with a *δ* Denotes the Reaction Energy Computed at the Given Level of Theory Subtracted from the Prior (e.g., *δ*CCSDTQ ≡ DLPNO–CCSDTQ – DLPNO–CCSDT­(Q))

HF	δMP2	δCCSD	δCCSD(T)	δCCSDT	δCCSDT(Q)	δCCSDTQ	Total
3.659	–4.682	+1.729	–0.500	+0.051	–0.054	–**0.097**	0.106

#### 5.3.2 (H_2_O)_17_ Water Cluster

Lastly, we compute conformation energies on the “sphere” and “552′5” conformations of a water cluster containing 17 water molecules. The geometries are derived from the work of Yoo and coworkers.^164^ Each system contains 68 active occupied orbitals, as well as a virtual space of 323 virtual orbitals. For these systems, the *T*_*CUT*_*XPNO*_ parameter (Table 4) is set to 10^–6^ to reduce possible projection error from the higher sparsity of water clusters and to help with convergence. Each CCSDTQ iteration took around 60 min in the 552′5 configuration and 75 min in the sphere configuration, taking up to 726 GB of RAM for the 552′5 configuration, and 800 GB for the spherical configuration. For these systems, we estimate a canonical CCSDTQ computation would take around 700,000 years per iteration (average of 4000 s per iteration, 16 natural orbitals out of 323, and 12000 quadruplets out of 967011 across the two systems). We present the energy difference between the 552′5 and sphere conformers (with the sphere being lower in energy) in Table 9. As expected, post-CCSD­(T) corrections have little impact on the conformation energy of water clusters, consistent with observations from prior high-level studies on smaller systems.^159^

**Table 9:** Energy Difference between Sphere and 552′5 Configurations of (H_2_O)_17_ ^164^ (Δ*E* = *E*_sphere_ – *E*_552′5_, kcal mol^–1^) at the cc-pVDZ Level of Theory, the Energy Difference in Each Column with a *δ* Denotes the Reaction Energy Computed at the Given Level of Theory Subtracted from the Prior (e.g., *δ*CCSDTQ ≡ DLPNO–CCSDTQ – DLPNO–CCSDT­(Q))

HF	δMP2	δCCSD	δCCSD(T)	δCCSDT	δCCSDT(Q)	δCCSDTQ	Total
–0.404	–2.832	+0.520	–0.353	+0.015	–0.016	–**0.001**	–3.071

#### 5.3.3 Adamantane

To showcase a computation involving a fully bonded 3D system, as opposed to molecular dimers, where there is inherently more sparsity, we present a computation on adamantane C_10_H_16_ on the cc-pVDZ basis (220 basis functions and 182 canonical virtual orbitals). The geometry, represented in Figure 6, was procured from the CCCBDB database from NIST,^165^ optimized at the MP2/cc-pVTZ level of theory, and will also be provided in the Supporting Information. In this computation, 11218 out of 30681 quadruplets survived the prescreening, and the average QNO rank is 15 (min of 8, max of 28), and every DLPNO–CCSDTQ iteration averaged around 9000 s per iteration. The computed (DLPNO) CCSDTQ-CCSDT­(Q) increment in absolute energies amounted to around −0.34 kcal mol^–1^ and the (DLPNO) CCSDTQ-CCSDT increment was −2.17 kcal mol^–1^ (full results in Supporting Information). In addition, around 850 GB of RAM was utilized. Without hardware optimizations or point group symmetry, we expect each iteration to take around 2500 years, using reasoning similar to that for the benzene dimer. In addition, the projected memory/hard drive requirements would scale as $O\left(o^{4}v^{4}\right)$ for the quadruples amplitudes, which means that the memory requirements would be around $\left(\frac{30681}{11218}\right)\times {\left(\frac{182}{15}\right)}^{4}$ or 60000 times greater, or around 50 petabytes! Since the number of retained QNOs is likely converged, and most of the quadruplets do not survive prescreening, we estimate that the rate-limiting factor for realistic applications of DLPNO–CCSDTQ would be the number of triplets $O\left(n^{3}\right)$ for a fully bonded 3D system. This is supported by the fact that none of the triplets have been prescreened for adamantane. Based on these estimates, with access to 3 TB of RAM, the current system size limit for a fully bonded 3D system is expected to be around 15 heavy atoms, or 40 total atoms, for DLPNO–CCSDTQ, with larger computations possible for nonfully bonded or non-3D systems.

**Figure 6:**
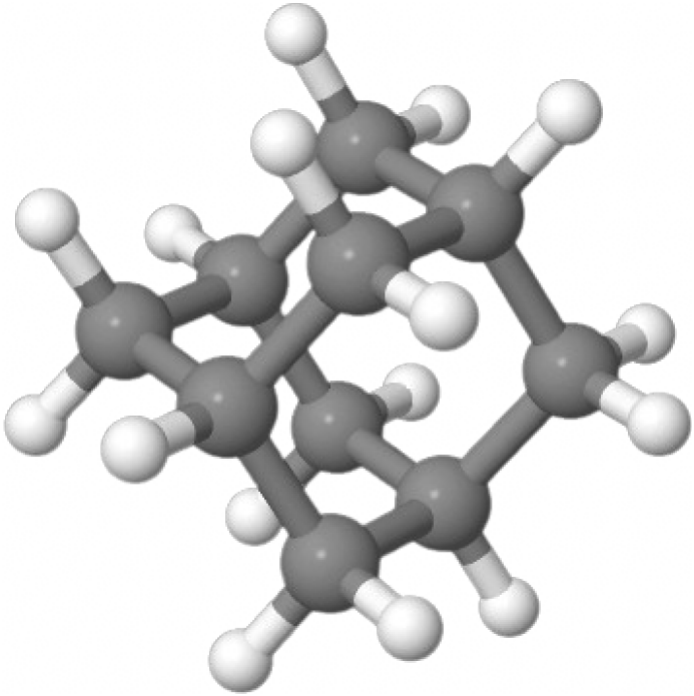
Geometry of adamantane used in this study (MP2/TZ).^165^

## 6 Conclusions

In this work, we present, to our knowledge, the first “local” linear-scaling coupled-cluster implementation that includes full quadruples excitations (also known as connected *T*_4_ contributions). Our method is convergent to canonical CCSDTQ and exactly captures subtle CCSDTQ-CCSDT­(Q) differences to a resolution on the order of 0.01–0.05 kcal mol^–1^, as shown with our tests on the HCNO reaction, the CH_2_OO enthalpy of formation, and the F_2_ + ClF → ClF_3_ reaction. We have also shown that it is possible to perform computations on much larger molecules than traditionally possible with CCSDTQ (4 heavy atoms or less), showcased by our computation on a benzene dimer and large water clusters. Based on prior experience with DLPNO–CCSD­(T), we expect the errors to not grow more than linearly with system size. Future accuracy benchmarks relative to canonical CCSDTQ, as well as DLPNO–CCSDTQ with tighter cutoffs, are encouraged as computing resources allow. We do, however, caution the use of our code on systems involving more than 5–6 heavy atoms (10–15 total), if users do not have access to more than 128 GB of RAM. Much of the errors from local approximations are encapsulated at the CCSD­(T) level of theory or lower, so a composite scheme such as DLPNO–CCSDTQ – DLPNO–CCSD­(T) + CCSD­(T) holds promise in making novel theoretical predictions, such as estimating the impact of conjugated π clouds on noncovalent interactions, a common motif in drug discovery projects. This is especially promising due to the widespread availability of efficient, canonical CCSD­(T) algorithms and our algorithm being implemented in the free and open-source Psi4 package. Other applications of our method can include the computation of Q-(Q) and CCSDTQ-CCSDT increments for larger molecules and basis sets in the W4 data set,^8^ and addressing the CCSD­(T) and FN-DMC controversy,^160,161,166,167^ where large discrepancies have been found between CCSD­(T) and FN-DMC results for noncovalent interaction energies with highly polarizable molecules, such as extended acenes. Though admittedly optimistic, with further optimization of our code through performance benchmarking and access to more powerful computers, we believe that DLPNO–CCSDTQ computations can be performed on some of the smaller systems with discrepancies in the near future. One of which is the 2D coronene dimer (72 atoms) from the L7 data set,^168^ where FN-DMC and CCSD­(T) results differ around 1.1 kcal mol^–1^ within error bars. Recent work by Lambie et al. suggests that CCSD­(T) does not overestimate interaction energies on extended acenes by computing CCSDT­(Q) and CCSDTQ contributions with a semiempirical Pariser–Parr–Pople (PPP) model Hamiltonian,^167^ and our method can be used to test and validate their claims. It is also noted that only CCSDT­(Q), and not CCSDTQ, computations were tractable with the model Hamiltonian in their work on the 2-dimensional acenes such as the coronene dimer, making such an application of our work even more impressive. Possible extensions of our work include an open-shell implementation of this algorithm,^110,169^ as well as the incorporation of explicit correlation.^170–172^ Despite the substantial computational resources necessary for the larger systems considered in this work, the favorable scalability of DLPNO–CCSDTQ allows these computations to be feasible. With continual advancements in computational hardware and increased availability of resources, such computations would become routine. Because our algorithm allows previously intractable computations to now be possible, we may say that this work represents a milestone in the future of computational quantum chemistry!

## Supplementary Material





## Data Availability

The data that support the findings of this study are available with the article and its Supporting Information. The Psi4 source code for DLPNO–CCSDTQ can be found at https://github.com/andyj10224/psi4/tree/full_q

## Notes

Geometries for systems studied, as well as absolute energies (ZIP)
Additional algorithmic information used for the computation of intermediates for DLPNO–CCSDTQ (PDF)

## References

[1] Crawford T. D., Schaefer H. F. (2000). An introduction to coupled cluster
theory for computational chemists. Rev. Comput.
Chem..

[2] Bartlett R. J., Musial M. (2007). Coupled-cluster theory in quantum chemistry. Rev. Mod. Phys..

[3] Sherrill C. D., Schaefer H. F. (1999). The Configuration Interaction Method:
Advances in Highly Correlated Approaches. Adv.
Quantum Chem..

[4] Cramer, C. J. Essentials of Computational Chemistry; John Wiley & Sons, 2002; pp. 191–232.

[5] Tajti A., Szalay P. G., Császár A. G., Kállay M., Gauss J., Valeev E. F., Flowers B. A., Vázquez J., Stanton J. F. (2004). HEAT: High accuracy extrapolated *ab initio* thermochemistry. J. Chem.
Phys..

[6] Bomble Y. J., Vázquez J., Kállay M., Michauk C., Szalay P. G., Császár A. G., Gauss J., Stanton J. F. (2006). High-accuracy
extrapolated ab initio thermochemistry. II. Minor improvements to
the protocol and a vital simplification. J.
Chem. Phys..

[7] Harding M. E., Vázquez J., Ruscic B., Wilson A. K., Gauss J., Stanton J. F. (2008). High-accuracy extrapolated ab initio thermochemistry.
III. Additional improvements and overview. J.
Chem. Phys..

[8] Karton A., Rabinovich E., Martin J. M. L., Ruscic B. (2006). W4 theory for computational
thermochemistry: In pursuit of confident sub-kJ/mol predictions. J. Chem. Phys.

[9] Karton A., Taylor P. R., Martin J. M. L. (2007). Basis
set convergence of post-CCSD
contributions to molecular atomization energies. J. Chem. Phys..

[10] Karton A., Martin J. M. L. (2010). Performance of
W4 theory for spectroscopic constants
and electrical properties of small molecules. J. Chem. Phys..

[11] Kucharski S. A., Bartlett R. J. (1992). The coupled–cluster
single, double, triple,
and quadruple excitation method. J. Chem. Phys..

[12] Oliphant N., Adamowicz L. (1991). Coupled–cluster
method truncated at quadruples. J. Chem. Phys..

[13] Kállay M., Surján P. R. (2001). Higher
excitations in coupled-cluster theory. J. Chem.
Phys..

[14] Kucharski S. A., Musiał M. (2010). Connected
quadruple excitations in the coupled-cluster
theory. Mol. Phys..

[15] Olsen J., Jørgensen P., Koch H., Balkova A., Bartlett R. J. (1996). Full configuration–interaction
and state of the art correlation calculations on water in a valence
double-zeta basis with polarization functions. J. Chem. Phys..

[16] Klippenstein S. J., Harding L. B., Ruscic B. (2017). Ab Initio
Computations and Active
Thermochemical Tables Hand in Hand: Heats of Formation of Core Combustion
Species. J. Phys. Chem. A.

[17] Elliott S. N., Keçeli M., Ghosh M. K., Somers K. P., Curran H. J., Klippenstein S. J. (2023). High-Accuracy
Heats of Formation for Alkane Oxidation:
From Small to Large via the Automated CBH-ANL Method. J. Phys. Chem. A.

[18] Puzzarini C. (2016). Accurate molecular
structures of small- and medium-sized molecules. Int. J. Quantum Chem..

[19] Puzzarini C. (2017). Astronomical
complex organic molecules: Quantum chemistry meets rotational spectroscopy. Int. J. Quantum Chem..

[20] Puzzarini C., Barone V. (2018). Diving for Accurate Structures in the Ocean of Molecular
Systems with the Help of Spectroscopy and Quantum Chemistry. Acc. Chem. Res..

[21] Puzzarini C., Stanton J. F. (2023). Connections between the accuracy
of rotational constants
and equilibrium molecular structures. Phys.
Chem. Chem. Phys..

[22] Puzzarini C., Ye H., Alessandrini S. (2024). Isomerism of CH2SO: Accurate structural, energetic,
and spectroscopic characterization. J. Comput.
Chem..

[23] Morgan W.
J., Matthews D. A., Ringholm M., Agarwal J., Gong J. Z., Ruud K., Allen W. D., Stanton J. F., Schaefer H. F. (2018). Geometric Energy Derivatives at the Complete Basis
Set Limit: Application to the Equilibrium Structure and Molecular
Force Field of Formaldehyde. J. Chem. Theory
Comput..

[24] Allen A. M., Sargent A. C., Schaefer H. F. (2025). Methylene: a turning point in the
history of quantum chemistry and an enduring paradigm. Pure Appl. Chem..

[25] Gauss J., Stanton J. F. (1995). Coupled–cluster calculations of nuclear magnetic
resonance chemical shifts. J. Chem. Phys..

[26] Gauss J., Stanton J. F. (1996). Perturbative treatment
of triple excitations in coupled–cluster
calculations of nuclear magnetic shielding constants. J. Chem. Phys..

[27] Rizzo A., Gauss J. (2002). Shielding polarizabilities calculated at the coupled-cluster singles
and doubles level augmented by a perturbative treatment of triple
excitations. J. Chem. Phys..

[28] Gauss J. (2002). Analytic second
derivatives for the full coupled-cluster singles, doubles, and triples
model: Nuclear magnetic shielding constants for BH, HF, CO, N2, N2O,
and O3. J. Chem. Phys..

[29] Lee Y. S., Kucharski S. A., Bartlett R. J. (1984). A coupled cluster approach with triple
excitations. J. Chem. Phys..

[30] Hoffmann M. R., Schaefer H. F. (1986). A Full Coupled-Cluster Singles, Doubles,
and Triples Model for the Description of Electron Correlation. Adv. Quantum Chem..

[31] Bartlett R. J., Watts J., Kucharski S., Noga J. (1990). Non-iterative fifth-order
triple and quadruple excitation energy corrections in correlated methods. Chem. Phys. Lett..

[32] Watts J. D., Cernusak I., Noga J., Bartlett R. J., Bauschlicher C. W., Lee T. J., Rendell A. P., Taylor P. R., Taylor P. R. (1990). Triple
and quadruple excitation contributions to the binding in Be clusters:
Calibration calculations on Be3. J. Chem. Phys..

[33] Scuseria G. E., Lee T. J. (1990). Comparison of coupled–cluster methods which
include the effects of connected triple excitations. J. Chem. Phys..

[34] Raghavachari K., Trucks G. W., Pople J. A., Head-Gordon M. (1989). A fifth-order
perturbation comparison of electron correlation theories. Chem. Phys. Lett..

[35] Watts J. D., Gauss J., Bartlett R. J. (1993). Coupled–cluster
methods with
noniterative triple excitations for restricted open–shell Hartree–Fock
and other general single determinant reference functions. Energies
and analytical gradients. J. Chem. Phys..

[36] Stanton J. F. (1997). Why CCSD­(T)
works: a different perspective. Chem. Phys.
Lett..

[37] Šimová L., Řezáč J., Hobza P. (2013). Convergence of the
Interaction Energies in Noncovalent Complexes in the Coupled-Cluster
Methods Up to Full Configuration Interaction. J. Chem. Theory Comput..

[38] Kodrycka M., Patkowski K. (2019). Platinum, gold, and silver standards of intermolecular
interaction energy calculations. J. Chem. Phys..

[39] Řezáč J., Šimová L., Hobza P. (2013). CCSD­[T] Describes Noncovalent Interactions
Better than the CCSD­(T), CCSD­(TQ), and CCSDT Methods. J. Chem. Theory Comput..

[40] Smith D. G. A., Jankowski P., Slawik M., Witek H. A., Patkowski K. (2014). Basis set
convergence of the post-CCSD­(T) contribution to noncovalent interaction
energies. J. Chem. Theory Comput..

[41] Karton A. (2019). Highly Accurate
CCSDT­(Q)/CBS Reaction Barrier Heights for a Diverse Set of Transition
Structures: Basis Set Convergence and Cost-Effective Approaches for
Estimating Post-CCSD­(T) Contributions. J. Phys.
Chem. A.

[42] Peterson K. A., Dunning T. H. J. (1995). Intrinsic Errors in Several ab Initio Methods: The
Dissociation Energy of N2. J. Phys. Chem..

[43] Bak K. L., Jørgensen P., Olsen J., Helgaker T., Klopper W. (2000). Accuracy of
atomization energies and reaction enthalpies in standard and extrapolated
electronic wave function/basis set calculations. J. Chem. Phys..

[44] Shirley W. A., Petersson G. (1991). The convergence of coupled-cluster methods for Be2. Chem. Phys. Lett..

[45] Kucharski S. A., Bartlett R. J. (1999). Connected quadruples for the frequencies
of O3. J. Chem. Phys..

[46] Feller D., Peterson K. A., Dixon D. A. (2012). Further
benchmarks of a composite,
convergent, statistically calibrated coupled-cluster-based approach
for thermochemical and spectroscopic studies. Mol. Phys..

[47] Martin J. M. (2014). The eight-valence-electron
systems re-examined: convergence of the coupled-cluster series and
performance of quasiperturbative methods for quadruple excitations. Mol. Phys..

[48] Feller D., Dixon D. A. (2003). Coupled Cluster Theory and Multireference
Configuration
Interaction Study of FO, F2O, FO2, and FOOF. J. Phys. Chem. A.

[49] Hopkins B. W., Tschumper G. S. (2004). Ab Initio Studies of *π*···*π* Interactions: The Effects of Quadruple Excitations. J. Phys. Chem. A.

[50] Klopper W., Bachorz R. A., Tew D. P., Aguilera-Iparraguirre J., Carissan Y., Hättig C. (2009). Accurate Coupled Cluster Calculations
of the Reaction Barrier Heights of Two CH3• + CH4 Reactions. J. Phys. Chem. A.

[51] Nguyen T. L., Li J., Dawes R., Stanton J. F., Guo H. (2013). Accurate Determination
of Barrier Height and Kinetics for the F + H2O → HF + OH Reaction. J. Phys. Chem. A.

[52] Matthews D. A., Stanton J. F. (2015). Non-orthogonal spin-adaptation of coupled cluster methods:
A new implementation of methods including quadruple excitations. J. Chem. Phys..

[53] Kucharski S. A., Bartlett R. J. (1998). An efficient way
to include connected quadruple contributions
into the coupled cluster method. J. Chem. Phys..

[54] Kucharski S. A., Bartlett R. J. (1998). Noniterative energy
corrections through fifth-order
to the coupled cluster singles and doubles method. J. Chem. Phys..

[55] Bomble Y. J., Stanton J. F., Kállay M., Gauss J. (2005). Coupled-cluster methods
including noniterative corrections for quadruple excitations. J. Chem. Phys..

[56] Kállay M., Gauss J. (2005). Approximate treatment
of higher excitations in coupled-cluster theory. J. Chem. Phys..

[57] Kállay M., Gauss J. (2008). Approximate treatment
of higher excitations in coupled-cluster theory.
II. Extension to general single-determinant reference functions and
improved approaches for the canonical Hartree-Fock case. J. Chem. Phys..

[58] Eriksen J. J., Jørgensen P., Olsen J., Gauss J. (2014). Equation-of-motion
coupled cluster perturbation theory revisited. J. Chem. Phys..

[59] Eriksen J. J., Kristensen K., Kjærgaard T., Jørgensen P., Gauss J. (2014). A Lagrangian framework
for deriving triples and quadruples corrections
to the CCSD energy. J. Chem. Phys..

[60] Eriksen J. J., Matthews D. A., Jørgensen P., Gauss J. (2015). Communication: The
performance of non-iterative coupled cluster quadruples models. J. Chem. Phys..

[61] Arponen J. (1983). Variational
principles and linked-cluster exp S expansions for static and dynamic
many-body problems. Ann. Phys..

[62] Adamowicz L., Laidig W. D., Bartlett R. J. (1984). Analytical
gradients for the coupled-cluster
method. Int. J. Quantum Chem..

[63] DePrince A. E., Sherrill C. D. (2013). Accuracy and Efficiency
of Coupled-Cluster Theory Using
Density Fitting/Cholesky Decomposition, Frozen Natural Orbitals, and
a *t*
_1_-Transformed Hamiltonian. J. Chem. Theory Comput..

[64] Gyevi-Nagy L., Kállay M., Nagy P. R. (2020). Integral-Direct and Parallel Implementation
of the CCSD­(T) Method: Algorithmic Developments and Large-Scale Applications. J. Chem. Theory Comput..

[65] Gyevi-Nagy L., Kállay M., Nagy P. R. (2021). Accurate Reduced-Cost
CCSD­(T) Energies:
Parallel Implementation, Benchmarks, and Large-Scale Applications. J. Chem. Theory Comput..

[66] Parrish R. M., Zhao Y., Hohenstein E. G., Martínez T. J. (2019). Rank reduced
coupled cluster theory. I. Ground state energies and wavefunctions. J. Chem. Phys..

[67] Hohenstein E. G., Zhao Y., Parrish R. M., Martínez T. J. (2019). Rank reduced
coupled cluster theory. II. Equation-of-motion coupled-cluster singles
and doubles. J. Chem. Phys..

[68] Lesiuk M. (2020). Implementation
of the Coupled-Cluster Method with Single, Double, and Triple Excitations
using Tensor Decompositions. J. Chem. Theory
Comput..

[69] Lesiuk M. (2021). Near-Exact
CCSDT Energetics from Rank-Reduced Formalism Supplemented by Non-iterative
Corrections. J. Chem. Theory Comput..

[70] Lesiuk M. (2022). When Gold
Is Not Enough: Platinum Standard of Quantum Chemistry with N7 Cost. J. Chem. Theory Comput..

[71] Parrish R. M., Hohenstein E. G., Martínez T. J., Sherrill C. D. (2012). Tensor hypercontraction.
II. Least-squares renormalization. J. Chem.
Phys..

[72] Hohenstein E. G., Parrish R. M., Martínez T. J. (2012). Tensor hypercontraction density fitting.
I. Quartic scaling second- and third-order Møller-Plesset perturbation
theory. J. Chem. Phys..

[73] Hohenstein E. G., Parrish R. M., Sherrill C. D., Martínez T. J. (2012). Communication:
Tensor hypercontraction. III. Least-squares tensor hypercontraction
for the determination of correlated wavefunctions. J. Chem. Phys..

[74] Parrish R. M., Sherrill C. D., Hohenstein E. G., Kokkila S. I. L., Martínez T. J. (2014). Communication:
Acceleration of coupled cluster singles and doubles via orbital-weighted
least-squares tensor hypercontraction. J. Chem.
Phys..

[75] Hohenstein E. G., Fales B. S., Parrish R. M., Martínez T. J. (2022). Rank-reduced
coupled-cluster. III. Tensor hypercontraction of the doubles amplitudes. J. Chem. Phys..

[76] Jiang A., Turney J. M., Schaefer H. F. (2023). Tensor Hypercontraction
Form of the Perturbative Triples Energy in Coupled-Cluster Theory. J. Chem. Theory Comput..

[77] Li S., Ma J., Jiang Y. (2002). Linear scaling local correlation approach for solving
the coupled cluster equations of large systems. J. Comput. Chem..

[78] Li S., Shen J., Li W., Jiang Y. (2006). An efficient implementation
of the “cluster-in-molecule” approach for local electron
correlation calculations. J. Chem. Phys..

[79] Li W., Piecuch P., Gour J. R., Li S. (2009). Local correlation calculations
using standard and renormalized coupled-cluster approaches. J. Chem. Phys..

[80] Li W., Piecuch P. (2010). Improved Design of
Orbital Domains within the Cluster-in-Molecule
Local Correlation Framework: Single-Environment Cluster-in-Molecule
Ansatz and Its Application to Local Coupled-Cluster Approach with
Singles and Doubles. J. Phys. Chem. A.

[81] Pulay P. (1983). Localizability
of dynamic electron correlation. Chem. Phys.
Lett..

[82] Schütz M., Hetzer G., Werner H.-J. (1999). Low-order scaling local electron
correlation methods. I. Linear scaling local MP2. J. Chem. Phys..

[83] Schütz M. (2000). Low-order
scaling local electron correlation methods. III. Linear scaling local
perturbative triples correction (T). J. Chem.
Phys..

[84] Schütz M., Werner H.-J. (2001). Low-order
scaling local electron correlation methods.
IV. Linear scaling local coupled-cluster (LCCSD). J. Chem. Phys..

[85] Schütz M. (2002). Low-order
scaling local electron correlation methods. V. Connected triples beyond
(T): Linear scaling local CCSDT-1b. J. Chem.
Phys..

[86] Schütz M., Manby F. R. (2003). Linear
scaling local coupled cluster theory with density
fitting. Part I: 4-external integrals. Phys.
Chem. Chem. Phys..

[87] Neogrády P., Pitoňák M., Urban M. (2005). Optimized virtual orbitals
for correlated calculations: an alternative approach. Mol. Phys..

[88] Taube A. G., Bartlett R. J. (2005). Frozen Natural Orbitals:
Systematic Basis Set Truncation
for Coupled-Cluster Theory. Collect. Czech.
Chem. Commun..

[89] Taube A. G., Bartlett R. J. (2008). Frozen natural orbital coupled-cluster theory: Forces
and application to decomposition of nitroethane. J. Chem. Phys..

[90] Rolik Z., Kállay M. (2011). A general-order
local coupled-cluster method based
on the cluster-in-molecule approach. J. Chem.
Phys..

[91] Rolik Z., Szegedy L., Ladjánszki I., Ladóczki B., Kállay M. (2013). An efficient linear-scaling CCSD­(T) method based on
local natural orbitals. J. Chem. Phys..

[92] Nagy P. R., Kállay M. (2017). Optimization of the linear-scaling local natural orbital
CCSD­(T) method: Redundancy-free triples correction using Laplace transform. J. Chem. Phys..

[93] Nagy P. R., Samu G., Kállay M. (2018). Optimization
of the Linear-Scaling
Local Natural Orbital CCSD­(T) Method: Improved Algorithm and Benchmark
Applications. J. Chem. Theory Comput..

[94] Neese F., Wennmohs F., Hansen A. (2009). Efficient
and accurate local approximations
to coupled-electron pair approaches: An attempt to revive the pair
natural orbital method. J. Chem. Phys..

[95] Neese F., Hansen A., Liakos D. G. (2009). Efficient and accurate approximations
to the local coupled cluster singles doubles method using a truncated
pair natural orbital basis. J. Chem. Phys..

[96] Riplinger C., Neese F. (2013). An efficient and near linear scaling pair natural orbital based local
coupled cluster method. J. Chem. Phys..

[97] Riplinger C., Sandhoefer B., Hansen A., Neese F. (2013). Natural triple excitations
in local coupled cluster calculations with pair natural orbitals. J. Chem. Phys..

[98] Liakos D. G., Sparta M., Kesharwani M. K., Martin J. M. L., Neese F. (2015). Exploring
the Accuracy Limits of Local Pair Natural Orbital Coupled-Cluster
Theory. J. Chem. Theory Comput..

[99] Werner H.-J., Knizia G., Krause C., Schwilk M., Dornbach M. (2015). Scalable Electron
Correlation Methods I.: PNO-LMP2 with Linear Scaling in the Molecular
Size and Near-Inverse-Linear Scaling in the Number of Processors. J. Chem. Theory Comput..

[100] Ma Q., Werner H.-J. (2015). Scalable Electron Correlation Methods. 2. Parallel
PNO-LMP2-F12 with Near Linear Scaling in the Molecular Size. J. Chem. Theory Comput..

[101] Pinski P., Riplinger C., Valeev E. F., Neese F. (2015). Sparse maps–A
systematic infrastructure for reduced-scaling electronic structure
methods. I. An efficient and simple linear scaling local MP2 method
that uses an intermediate basis of pair natural orbitals. J. Chem. Phys..

[102] Riplinger C., Pinski P., Becker U., Valeev E. F., Neese F. (2016). Sparse maps–A systematic infrastructure
for reduced-scaling
electronic structure methods. II. Linear scaling domain based pair
natural orbital coupled cluster theory. J. Chem.
Phys..

[103] Schwilk M., Ma Q., Köppl C., Werner H.-J. (2017). Scalable Electron Correlation Methods. 3. Efficient
and Accurate Parallel Local Coupled Cluster with Pair Natural Orbitals
(PNO-LCCSD). J. Chem. Theory Comput..

[104] Ma Q., Schwilk M., Köppl C., Werner H.-J. (2017). Scalable Electron
Correlation Methods. 4. Parallel Explicitly Correlated Local Coupled
Cluster with Pair Natural Orbitals (PNO-LCCSD-F12). J. Chem. Theory Comput..

[105] Guo Y., Riplinger C., Becker U., Liakos D. G., Minenkov Y., Cavallo L., Neese F. (2018). Communication: An improved
linear
scaling perturbative triples correction for the domain based local
pair-natural orbital based singles and doubles coupled cluster method
[DLPNO-CCSD­(T)]. J. Chem. Phys..

[106] Ma Q., Werner H.-J. (2018). Scalable Electron Correlation Methods. 5. Parallel
Perturbative Triples Correction for Explicitly Correlated Local Coupled
Cluster with Pair Natural Orbitals. J. Chem.
Theory Comput..

[107] Jiang A., Glick Z. L., Poole D., Turney J. M., Sherrill C. D., Schaefer H. F. (2024). Accurate and
efficient open-source implementation of domain-based local pair natural
orbital (DLPNO) coupled-cluster theory using a t1-transformed Hamiltonian. J. Chem. Phys.

[108] Jiang A., Schaefer H. F., Turney J. M. (2025). Linear-Scaling
Local Natural Orbital-Based Full Triples Treatment in Coupled-Cluster
Theory. J. Chem. Theory Comput..

[109] Jiang A., Schaefer H. F., Turney J. M. (2025). Linear-scaling
quadruple excitations in local pair natural orbital coupled-cluster
theory. J. Chem. Phys..

[110] Saitow M., Becker U., Riplinger C., Valeev E. F., Neese F. (2017). A new near-linear scaling, efficient
and accurate, open-shell domain-based local pair natural orbital coupled
cluster singles and doubles theory. J. Chem.
Phys..

[111] Nagy P. R., Kállay M. (2019). Approaching the Basis Set Limit of
CCSD­(T) Energies for Large Molecules with Local Natural Orbital Coupled-Cluster
Methods. J. Chem. Theory Comput..

[112] Fishman, V. ; Lörincz, B. D. ; Semidalas, E. ; Barman, A. ; Martin, J. M. L. ; Nagy, P. R. ; Kállay, M. ; Development of local natural orbital arbitrary order coupled cluster methods and assessment through connected quadruples. 2026, 10.26434/chemrxiv.10001857/v1. PMC13112354 41949447

[113] Martin, J. M. L. ; Semidalas, E. How ‘Nonvariational’ Are Approximate Coupled Cluster Methods In Practice?. arXiv:2410.01358. 2024.

[114] Begley J. M., Aroeira G. J. R., Turney J. M., Douberly G. E., Schaefer H. F. (2023). Enthalpies of formation for Criegee
intermediates: A correlation energy convergence study. J. Chem. Phys..

[115] Allen A. M., Olive L. N., Gonzalez
Franco P. A., Barua S. R., Allen W. D., Schaefer H. F. (2024). Fulminic acid: a quasibent spectacle. Phys.
Chem. Chem. Phys..

[116] Spiegel M., Semidalas E., Martin J. M. L., Bentley M. R., Stanton J. F. (2024). Post-CCSD­(T)
corrections to bond distances and vibrational
frequencies: the power of Λ. Mol. Phys..

[117] Koch H., Christiansen O., Kobayashi R., Jørgensen P., Helgaker T. (1994). A direct atomic orbital
driven implementation
of the coupled cluster singles and doubles (CCSD) model. Chem. Phys. Lett..

[118] Whitten J. L. (1973). Coulombic potential energy integrals and approximations. J. Chem. Phys..

[119] Feyereisen M., Fitzgerald G., Komornicki A. (1993). Use of approximate
integrals in ab initio theory. An application in MP2 energy calculations. Chem. Phys. Lett..

[120] Dunlap B. I., Connolly J. W. D., Sabin J. R. (1979). On some approximations
in applications of X*α* theory. J. Chem. Phys..

[121] Vahtras O., Almlöf J., Feyereisen M. (1993). Integral approximations
for LCAO-SCF calculations. Chem. Phys. Lett..

[122] Rendell A. P., Lee T. J. (1994). Coupled–cluster
theory employing
approximate integrals: An approach to avoid the input/output and storage
bottlenecks. J. Chem. Phys..

[123] Weigend F., Häser M., Patzelt H., Ahlrichs R. (1998). RI-MP2: optimized
auxiliary basis sets and demonstration of efficiency. Chem. Phys. Lett..

[124] Weigend F. (2002). A fully direct RI-HF algorithm: Implementation, optimized
auxiliary basis sets, demonstration of accuracy and efficiency. Phys. Chem. Chem. Phys..

[125] Sodt A., Subotnik J. E., Head-Gordon M. (2006). Linear scaling
density fitting. J. Chem. Phys..

[126] Werner H.-J., Manby F. R., Knowles P. J. (2003). Fast linear scaling
second-order Møller-Plesset perturbation theory (MP2) using local
and density fitting approximations. J. Chem.
Phys..

[127] Beebe N.
H. F., Linderberg J. (1977). Simplifications
in the generation
and transformation of two-electron integrals in molecular calculations. Int. J. Quantum Chem..

[128] Røeggen I., Johansen T. (2008). Cholesky decomposition of the two-electron
integral matrix in electronic structure calculations. J. Chem. Phys..

[129] Pedersen T. B., Lehtola S., Galván I. F., Lindh R. (2024). The versatility of the Cholesky decomposition in electronic structure
theory. Wiley Interdiscip. Rev. Comput. Mol.
Sci..

[130] Jensen, F. Introduction to Computational Chemistry; John wiley & sons, 2007; pp. 204–208.

[131] Foster J. M., Boys S. F. (1960). Canonical Configurational
Interaction
Procedure. Rev. Mod. Phys..

[132] Boughton J. W., Pulay P. (1993). Comparison of the boys
and Pipek-Mezey
localizations in the local correlation approach and automatic virtual
basis selection. J. Comput. Chem..

[133] Pipek J., Mezey P. G. (1989). A fast intrinsic
localization procedure
applicable for ab–initio and semiempirical linear combination
of atomic orbital wave functions. J. Chem. Phys..

[134] Edmiston C., Ruedenberg K. (1963). Localized
Atomic and Molecular Orbitals. Rev. Mod. Phys..

[135] Folkestad S. D., Matveeva R., Høyvik I.-M., Koch H. (2022). Implementation of Occupied and Virtual Edmiston–Ruedenberg
Orbitals Using Cholesky Decomposed Integrals. J. Chem. Theory Comput..

[136] Mulliken R. S. (1955). Electronic Population Analysis on LCAO–MO Molecular
Wave Functions. I. J. Chem. Phys..

[137] Häser M., Ahlrichs R. (1989). Improvements on the
direct SCF method. J. Comput. Chem..

[138] Edmiston C., Krauss M. (1965). Configuration–Interaction
Calculation of H3 and H2. J. Chem. Phys..

[139] Meyer W. (1971). Ionization energies of water from
PNO-CI calculations. Int. J. Quantum Chem..

[140] Meyer W. (1973). PNO–CI Studies of electron
correlation effects. I. Configuration
expansion by means of nonorthogonal orbitals, and application to the
ground state and ionized states of methane. J. Chem. Phys..

[141] Ahlrichs R., Driessler F., Lischka H., Staemmler V., Kutzelnigg W. (1975). PNO–CI (pair natural orbital configuration interaction)
and CEPA–PNO (coupled electron pair approximation with pair
natural orbitals) calculations of molecular systems. II. The molecules
BeH2, BH, BH3, CH4, CH–3, NH3 (planar and pyramidal), H2O,
OH+3, HF and the Ne atom. J. Chem. Phys..

[142] Taylor P. R., Bacskay G., Hush N., Hurley A. (1976). The coupled-pair
approximation in a basis of independent-pair natural orbitals. Chem. Phys. Lett..

[143] Dykstra C. E., Schaefer H. F., Meyer W. (1976). A theory of
self–consistent electron pairs. Computational methods and preliminary
applications. J. Chem. Phys..

[144] Møller C., Plesset M. S. (1934). Note on an Approximation
Treatment
for Many-Electron Systems. Phys. Rev..

[145] Kolda T. G., Bader B. W. (2009). Tensor Decompositions
and Applications. SIAM Rev..

[146] Lesiuk M. (2022). Quintic-scaling rank-reduced coupled
cluster theory
with single and double excitations. J. Chem.
Phys..

[147] Neese F., Wennmohs F., Becker U., Riplinger C. (2020). The ORCA quantum
chemistry program package. J. Chem. Phys..

[148] Pulay P. (1980). Convergence
acceleration of iterative sequences. the case of scf
iteration. Chem. Phys. Lett..

[149] Scuseria G. E., Lee T. J., Schaefer H. F. (1986). Accelerating the convergence of the coupled-cluster
approach: The
use of the DIIS method. Chem. Phys. Lett..

[150] Matthews D. A., Stanton J. F. (2015). Accelerating the
convergence of higher-order
coupled cluster methods. J. Chem. Phys..

[151] Matthews D. A. (2020). Accelerating
the convergence of higher-order coupled-cluster
methods II: coupled-cluster Λ equations and dynamic damping. Mol. Phys..

[152] Smith D. G. A. (2018). Psi4NumPy: An Interactive Quantum Chemistry
Programming Environment for Reference Implementations and Rapid Development. J. Chem. Theory Comput..

[153] Matthews D. A., Cheng L., Harding M. E., Lipparini F., Stopkowicz S., Jagau T.-C., Szalay P. G., Gauss J., Stanton J. F. (2020). Coupled-cluster techniques for computational chemistry:
The CFOUR program package. J. Chem. Phys..

[154] Smith D. G. A., Burns L. A., Simmonett A. C., Parrish R. M., Schieber M. C., Galvelis R., Kraus P., Kruse H., Di Remigio R., Alenaizan A. (2020). Psi4 1.4: Open-source software for high-throughput
quantum chemistry. J. Chem. Phys..

[155] Turney, J. ; Briggs, C. Einsums v 0.6.1, 2024. https://github.com/Einsums/Einsums.

[156] Active Thermochemical Tables (ATcT) https://atct.anl.gov, 2026.

[157] Karton A., Sylvetsky N., Martin J. M. L. (2017). W4–17:
A diverse and high-confidence dataset of atomization energies for
benchmarking high-level electronic structure methods. J. Comput. Chem..

[158] Marshall M. S., Burns L. A., Sherrill C. D. (2011). Basis set convergence
of the coupled-cluster correction, : Best practices for benchmarking
non-covalent interactions and the attendant revision of the S22, NBC10,
HBC6, and HSG databases. J. Chem. Phys..

[159] Semidalas E., Boese A. D., Martin J. M. (2025). Post-CCSD­(T) corrections
in the S66 noncovalent interactions benchmark. Chem. Phys. Lett..

[160] Villot C., Ballesteros F., Wang D., Lao K. U. (2022). Coupled
Cluster Benchmarking of Large Noncovalent Complexes in L7 and S12L
as Well as the C60 Dimer, DNA–Ellipticine, and HIV–Indinavir. J. Phys. Chem. A.

[161] Schäfer T., Irmler A., Gallo A., Grüneis A. (2025). Understanding
discrepancies in noncovalent interaction energies from wavefunction
theories for large molecules. Nature Comm..

[162] Karton A. (2019). Basis set convergence of high-order coupled cluster
methods up to CCSDTQ567 for a highly multireference molecule. Chem. Phys. Lett..

[163] Karton A. (2020). Effective
basis set extrapolations for CCSDT, CCSDT­(Q),
and CCSDTQ correlation energies. J. Chem. Phys..

[164] Yoo S., Aprà E., Zeng X. C., Xantheas S. S. (2010). High-Level Ab Initio
Electronic Structure Calculations of Water Clusters (H2O)­16 and (H2O)­17:
A New Global Minimum for (H2O)­16. J. Phys. Chem.
Lett..

[165] Computational Chemistry Comparison and Benchmark DataBase (CCCBDB). https://cccbdb.nist.gov/, 2026.

[166] Al-Hamdani Y. S., Nagy P. R., Zen A., Barton D., Kállay M., Brandenburg J. G., Tkatchenko A. (2021). Interactions
between large molecules pose a puzzle for reference quantum mechanical
methods. Nature Comm..

[167] Lambie S., Kats D., Usvyat D., Alavi A. (2025). On the applicability
of CCSD­(T) for dispersion interactions in large conjugated systems. J. Chem. Phys..

[168] Sedlak R., Janowski T., Pitoňák M., Řezáč J., Pulay P., Hobza P. (2013). Accuracy of
Quantum Chemical Methods for Large Noncovalent Complexes. J. Chem. Theory Comput..

[169] Guo Y., Riplinger C., Liakos D. G., Becker U., Saitow M., Neese F. (2020). Linear scaling perturbative triples correction approximations for
open-shell domain-based local pair natural orbital coupled cluster
singles and doubles theory [DLPNO-CCSD­(T/T)]. J. Chem. Phys..

[170] Pavošević F., Pinski P., Riplinger C., Neese F., Valeev E. F. (2016). SparseMaps–A
systematic infrastructure
for reduced-scaling electronic structure methods. IV. Linear-scaling
second-order explicitly correlated energy with pair natural orbitals. J. Chem. Phys.

[171] Pavošević F., Peng C., Pinski P., Riplinger C., Neese F., Valeev E. F. (2017). SparseMaps–A
systematic infrastructure for reduced scaling electronic structure
methods. V. Linear scaling explicitly correlated coupled-cluster method
with pair natural orbitals. J. Chem. Phys..

[172] Mitchell E. C., Turney J. M., Schaefer H. F. (2024). Automatic Differentiation
for Explicitly Correlated MP2. J. Chem. Theory
Comput..

