# Supplementary material for: Local Pair Natural Orbital-Based Coupled-Cluster Theory through Full Quadruples (DLPNO–CCSDTQ)
Source: J Chem Theory Comput. 2026 Mar 6;22(6):2825–45. doi: 10.1021/acs.jctc.5c01910 (PMC13019630; doi:10.1021/acs.jctc.5c01910)
Supplement: Supplementary file 2 [file ct5c01910_si_002.pdf]

Supplemental File For: Local Pair Natural Orbital-based Coupled-Cluster Theory through Full Quadruples (DLPNO-CCSDTQ)

Andy Jiang,[1] Devin A. Matthews,[2] David Poole,[3] Connor G. Briggs,[1] Justin M. Turney,[1] C. David Sherrill,[3] and Henry F. Schaefer III[1,*]

[1]*Center for Computational Quantum Chemistry, Department of Chemistry, University of Georgia, Athens, GA 30602, United States of America*

[2]*Department of Chemistry, Southern Methodist University, Dallas, Texas 75275, United States of America*

[3]*Center for Computational Molecular Science and Technology, School of Chemistry and Biochemistry, School of Computational Science and Engineering, Georgia Institute of Technology, Atlanta, GA 30332-0400*

* ccq@uga.edu

SI. EQUATIONS FOR R_{ijkl}^{abcd} INTERMEDIATES

Most of these terms can be derived in a straightforward manner, with a couple of exceptions. All intermediates are expressed in the domain of $ijkl$, and all integrals are taken from the domain of $ijkl$, with the amplitudes in each contraction projected from its corresponding space to the QNO domain of $ijkl$.

$$\begin{aligned} A_{ej}^{ab}[ijkl] = (\widetilde{B}_{ae}^{Q}\widetilde{B}_{bj}^{Q})[ijkl] + (B_{me}^{Q}\widetilde{B}_{nj}^{Q})[ijkl] \cdot T_{mn}^{ab}(|a_{mn}\rangle \to |a_{ijkl}\rangle) + \frac{1}{2}(2B_{mf}^{Q}\widetilde{B}_{ae}^{Q} - \widetilde{B}_{af}^{Q}B_{me}^{Q})[ijkl] \\ \times U_{mj}^{fb}(|a_{mj}\rangle \to |a_{ijkl}\rangle) - (\frac{1}{2} + P_{ab})[(B_{me}^{Q}\widetilde{B}_{af}^{Q})[ijkl] \cdot T_{jm}^{fb}(|a_{jm}\rangle \to |a_{ijkl}\rangle)] \\ - (B_{me}^{Q}B_{nf}^{Q})[ijkl] \cdot Z_{nmj}^{fab}(|a_{nmj}\rangle \to |a_{ijkl}\rangle) - \widetilde{F}_{me}[ijkl] \cdot T_{mj}^{ab}(|a_{mj}\rangle \to |a_{ijkl}\rangle) \ , \end{aligned} \tag{S1}$$

$$\begin{aligned} B_{ij}^{am}[ijkl] = (\widetilde{B}_{ai}^{Q}\widetilde{B}_{mj}^{Q})[ijkl] + (\widetilde{B}_{ae}^{Q}B_{mf}^{Q})[ijkl] \cdot T_{ij}^{ef}(|a_{ij}\rangle \to |a_{ijkl}\rangle) \\ + \frac{1}{2}(2B_{ne}^{Q}\widetilde{B}_{mj}^{Q} - \widetilde{B}_{nj}^{Q}B_{me}^{Q})[ijkl] \cdot U_{ni}^{ea}(|a_{ni}\rangle \to |a_{ijkl}\rangle) - \frac{1}{2}(\widetilde{B}_{nj}^{Q}B_{me}^{Q})[ijkl] \cdot T_{in}^{ea}(|a_{in}\rangle \to |a_{ijkl}\rangle) \\ - (\widetilde{B}_{ni}^{Q}B_{me}^{Q})[ijkl] \cdot T_{jn}^{ea}(|a_{jn}\rangle \to |a_{ijkl}\rangle) + \widetilde{F}_{me}[ijkl] \cdot T_{ij}^{ae}(|a_{ij}\rangle \to |a_{ijkl}\rangle) \\ + (B_{me}^{Q}B_{nf}^{Q})[ijkl] \cdot Z_{nij}^{fae}(|a_{nij}\rangle \to |a_{ijkl}\rangle) \ , \end{aligned} \tag{S2}$$

$$\widetilde{\widetilde{F}}_{ae}[ijkl] = \widetilde{F}_{ae}[ijkl] - (2B_{nf}^{Q}B_{me}^{Q} - B_{ne}^{Q}B_{mf}^{Q})[ijkl] \cdot T_{nm}^{fa}(|a_{nm}\rangle \to |a_{ijkl}\rangle) \ , \tag{S3}$$

$$\widetilde{\widetilde{F}}_{mi}[ijkl] = \widetilde{F}_{mi}[ijkl] + (2B_{nf}^{Q}B_{me}^{Q} - B_{ne}^{Q}B_{mf}^{Q})[ijkl] \cdot T_{ni}^{fe}(|a_{ni}\rangle \to |a_{ijkl}\rangle) \ , \tag{S4}$$

$$E_{ei}^{ma}[ijkl] = (2B_{me}^{Q}\widetilde{B}_{ai}^{Q} - \widetilde{B}_{mi}^{Q}\widetilde{B}_{ae}^{Q})[ijkl] + (2B_{nf}^{Q}B_{me}^{Q} - B_{ne}^{Q}B_{mf}^{Q})[ijkl] \cdot U_{ni}^{fa}(|a_{ni}\rangle \to |a_{ijkl}\rangle) \ , \tag{S5}$$

$$F_{ie}^{ma}[ijkl] = (\widetilde{B}_{mi}^{Q}\widetilde{B}_{ae}^{Q})[ijkl] - (B_{ne}^{Q}B_{mf}^{Q})[ijkl] \cdot T_{in}^{fa}(|a_{in}\rangle \to |a_{ijkl}\rangle) \ , \tag{S6}$$

$$G_{ij}^{mn}[ijkl] = (\widetilde{B}_{mi}^{Q}\widetilde{B}_{nj}^{Q})[ijkl] + (B_{me}^{Q}B_{nf}^{Q})[ijkl] \cdot T_{ij}^{ef}(|a_{ij}\rangle \to |a_{ijkl}\rangle) \ , \tag{S7}$$

$$H_{ef}^{ab}[ijkl] = (\widetilde{B}_{ae}^{Q}\widetilde{B}_{bf}^{Q})[ijkl] + (B_{me}^{Q}B_{nf}^{Q})[ijkl] \cdot T_{mn}^{ab}(|a_{mn}\rangle \to |a_{ijkl}\rangle) \ , \tag{S8}$$

$$I_{eij}^{mab}[ijkl] = P_{ij}^{ab}[(2B_{me}^{Q}\widetilde{B}_{af}^{Q} - B_{mf}^{Q}\widetilde{B}_{ae}^{Q})[ijkl] \cdot T_{ji}^{bf}(|a_{ji}\rangle \to |a_{ijkl}\rangle) - (2B_{me}^{Q}\widetilde{B}_{ni}^{Q} - \widetilde{B}_{mi}^{Q}B_{ne}^{Q})[ijkl] \cdot T_{nj}^{ab}(|a_{nj}\rangle \to |a_{ijkl}\rangle) + \frac{1}{4}(2B_{nf}^{Q}B_{me}^{Q} - B_{ne}^{Q}B_{mf}^{Q})[ijkl] \cdot Z_{nij}^{fab}(|a_{nij}\rangle \to |a_{ijkl}\rangle)] \ , \quad \text{(S9)}$$

$$J_{iej}^{mab}[ijkl] = (B_{mf}^{Q}\widetilde{B}_{ae}^{Q})[ijkl] \cdot T_{ji}^{bf}(|a_{ji}\rangle \to |a_{ijkl}\rangle) - (\widetilde{B}_{mi}^{Q}B_{ne}^{Q})[ijkl] \cdot T_{nj}^{ab}(|a_{nj}\rangle \to |a_{ijkl}\rangle) - \frac{1}{2}(B_{ne}^{Q}B_{mf}^{Q})[ijkl] \cdot T_{inj}^{fab}(|a_{inj}\rangle \to |a_{ijkl}\rangle) \ , \quad \text{(S10)}$$

$$K_{ijk}^{amn}[ijkl] = P_{jk}^{mn}[(B_{me}^{Q}\widetilde{B}_{nk}^{Q})[ijkl] \cdot T_{ij}^{ae}(|a_{ij}\rangle \to |a_{ijkl}\rangle) + \frac{1}{2}(B_{me}^{Q}B_{nf}^{Q})[ijkl] \cdot T_{ijk}^{aef}(|a_{ijk}\rangle \to |a_{ijkl}\rangle)] \ , \quad \text{(S11)}$$

These last two terms represent exceptions, with some of these contractions initially represented in PNO spaces to reduce the cost of the evaluations (More details in Section IIIB of the main text).

$$L_{ijk}^{abm}[ijkl] = P_{ij}^{ab}[(\widetilde{B}_{ae}^{Q}B_{mf}^{Q})[ijkl] \cdot T_{ijk}^{ebf}(|a_{ijk}\rangle \to |a_{ijkl}\rangle) + \frac{1}{2}(E_{ei}^{ma} + F_{ie}^{ma})[ijkl] \cdot T_{jk}^{be}(|a_{jk}\rangle \to |a_{ijkl}\rangle) + F_{ke}^{ma}[ijkl] \cdot T_{ji}^{be}(|a_{ji}\rangle \to |a_{ijkl}\rangle) - \frac{1}{2}G_{ki}^{mn}[ijkl] \cdot T_{nj}^{ab}(|a_{nj}\rangle \to |a_{ijkl}\rangle) + \delta L_{ijk}^{abm}[ij](|a_{ij}\rangle \to |a_{ijkl}\rangle)] \ , \quad \text{(S12)}$$

$$\delta L_{ijk}^{abm}[ij] = \frac{1}{2}(B_{me}^{Q}B_{nf}^{Q})[ij] \cdot \alpha_{nijk}^{fabe}(|a_{nijk}\rangle \to |a_{ij}\rangle) \ . \quad \text{(S13)}$$

$$M_{ejk}^{abc}[ijkl] = P_{jk}^{bc}[\frac{1}{2}H_{ef}^{ab}[ijkl] \cdot T_{jk}^{fc}(|a_{jk}\rangle \to |a_{ijkl}\rangle) + \delta M_{ejk}^{abc}[jk](|a_{jk}\rangle \to |a_{ijkl}\rangle)] \ , \quad \text{(S14)}$$

$$\delta M_{ejk}^{abc}[jk] = -\frac{1}{2}(B_{me}^{Q}B_{nf}^{Q})[jk] \cdot \alpha_{nmjk}^{fabc}(|a_{nmjk}\rangle \to |a_{jk}\rangle) \ . \quad \text{(S15)}$$

SII. CONTRIBUTION TO R_{ij}^{ab}

In these next three sections, working equations that fully take advantage of symmetry and reduce the number of tensor contractions that need to be performed are presented, as currently implemented in the code. For this term, the $B_{me}^{Q}B_{nf}^{Q}$ is computed in the domain of $mnij$, contracted with β_{mnij}^{efab}, and the resulting product is projected onto the PNO space of ij before being summed over.

$$R_{ij}^{ab} \mathrel{+}= \frac{1}{2}B_{me}^{Q}B_{nf}^{Q}\beta_{mnij}^{efab} \; . \tag{S16}$$

SIII. CONTRIBUTIONS TO R_{ijk}^{abc}

The localized virtual space projection/contraction patterns for these are given explicitly in Section IIIB in the manuscript.

A. $\widetilde{F}_{me}$ contribution

$$R_{ijk}^{abc} \mathrel{+}= \widetilde{F}_{me}\alpha_{mijk}^{eabc} \; . \tag{S17}$$

B. $(ae\widetilde{|}mf)$ contribution

$$R_{ijk}^{abc} \mathrel{+}= (\widetilde{B}_{ae}^{Q}B_{mf}^{Q}\alpha_{mijk}^{febc} + \widetilde{B}_{be}^{Q}B_{mf}^{Q}\alpha_{mjik}^{feac} + \widetilde{B}_{ce}^{Q}B_{mf}^{Q}\alpha_{mkij}^{feab}) \; . \tag{S18}$$

C. $(me\widetilde{|}nj)$ contribution

$$R_{ijk}^{abc} \mathrel{-}= (B_{me}^{Q}\widetilde{B}_{ni}^{Q}\alpha_{mnjk}^{eabc} + B_{me}^{Q}\widetilde{B}_{nj}^{Q}\alpha_{mnik}^{ebac} + B_{me}^{Q}\widetilde{B}_{nk}^{Q}\alpha_{mnij}^{ecab}) \; . \tag{S19}$$

SIV. CONTRIBUTIONS TO R^{abcd}_{ijkl}

The symmetrized forms of the quadruples amplitude contributions are presented in this section. Deriving the forms of these equations in the localized space is straightforward, as all these quantities involve an intermediate computed in the domain of $ijkl$ (Section SII) contracted with amplitudes (projected from their corresponding spaces to the QNO space of $ijkl$).

A. A^{ab}_{ej} contribution

$$R^{abcd}_{ijkl} \mathrel{+}= \sum_{\binom{ijkl}{i'j'/k'l'}} P_{(i',j',k',l')}[A^{ab}_{ej'}T^{ecd}_{i'k'l'} + A^{ba}_{ei'}T^{ecd}_{j'k'l'}] \ , \tag{S20}$$

where $P_{(i,j,k,l)}$ permutes the virtual space indices as defined in Section IIIC in the manuscript, and the notation $\binom{ijkl}{i'j'/k'l'}$ represents (i',j') being selected from the pair list $\{(i,j),(i,k),(i,l),(j,k),(j,l),(k,l)\}$ and (k',l') representing the complement– e.g, if $(i',j') = (j,l)$, then $(k',l') = (i,k)$.

B. B^{am}_{ij} contribution

Similar to the A^{ab}_{ej} contribution,

$$R^{abcd}_{ijkl} \mathrel{-}= \sum_{\binom{ijkl}{i'j'/k'l'}} P_{(i',j',k',l')}[B^{am}_{i'j'}T^{bcd}_{mk'l'} + B^{bm}_{j'i'}T^{acd}_{mk'l'}] \ . \tag{S21}$$

C. $\widetilde{\widetilde{F}}_{ae}$ contribution

$$R^{abcd}_{ijkl} \mathrel{+}= \sum_{\binom{ijkl}{i'/j'k'l'}} P_{(i',j',k',l')}[\widetilde{\widetilde{F}}_{ae}T^{ebcd}_{i'j'k'l'}] \ . \tag{S22}$$

where this expression uses a similar notation $\binom{ijkl}{i'/j'k'l'}$, with i' representing a single element out of (i,j,k,l) and $j'k'l'$ representing the list of three non-selected elements– e.g. if $i' = k$, then $j'k'l' = ijl$. Note that this is equivalent to:

$$R_{ijkl}^{abcd} \mathrel{+}= \widetilde{\widetilde{F}}_{ae} T_{ijkl}^{ebcd} + \widetilde{\widetilde{F}}_{be} T_{ijkl}^{aecd} + \widetilde{\widetilde{F}}_{ce} T_{ijkl}^{abed} + \widetilde{\widetilde{F}}_{de} T_{ijkl}^{abce} \ . \tag{S23}$$

D. $\widetilde{\widetilde{F}}_{mi}$ contribution

$$R_{ijkl}^{abcd} \mathrel{-}= \sum_{\binom{ijkl}{i'/j'k'l'}} P_{(i',j',k',l')} [\widetilde{\widetilde{F}}_{mi'} T_{mj'k'l'}^{abcd}] \ . \tag{S24}$$

This is equivalent to

$$R_{ijkl}^{abcd} \mathrel{-}= (\widetilde{\widetilde{F}}_{mi} T_{mjkl}^{abcd} + \widetilde{\widetilde{F}}_{mj} T_{imkl}^{abcd} + \widetilde{\widetilde{F}}_{mk} T_{ijml}^{abcd} + \widetilde{\widetilde{F}}_{ml} T_{ijkm}^{abcd}) \ . \tag{S25}$$

E. E_{ei}^{ma} contribution

$$R_{ijkl}^{abcd} \mathrel{+}= \sum_{\binom{ijkl}{i'/j'k'l'}} \frac{1}{2} P_{(i',j',k',l')} [E_{ei'}^{ma} \alpha_{mj'k'l'}^{ebcd}] \ . \tag{S26}$$

F. F_{ie}^{ma} contribution

$$R_{ijkl}^{abcd} \mathrel{-}= \sum_{\binom{ijkl}{i'j'/k'l'}} (\frac{1}{2} + P_{ab}) [F_{i'e}^{ma} T_{j'mk'l'}^{ebcd} + F_{j'e}^{mb} T_{i'mk'l'}^{eacd}] \ . \tag{S27}$$

G. G_{ij}^{mn} contribution

$$R_{ijkl}^{abcd} \mathrel{+}= \sum_{\binom{ijkl}{i'j'/k'l'}} P_{(i',j',k',l')} [G_{i'j'}^{mn} T_{mnk'l'}^{abcd}] \ . \tag{S28}$$

H. H_{ef}^{ab} contribution

$$R_{ijkl}^{abcd} \mathrel{+}= \sum_{\binom{ijkl}{i'j'/k'l'}} P_{(i',j',k',l')} [H_{ef}^{ab} T_{i'j'k'l'}^{efcd}] \ . \tag{S29}$$

I. I^{mab}_{eij} contribution

$$R^{abcd}_{ijkl} \mathrel{+}= \sum_{\binom{ijkl}{i'j'/k'l'}} \frac{1}{2} P_{(i',j',k',l')}[I^{mab}_{ei'j'} Z^{ecd}_{mk'l'}] \ . \tag{S30}$$

J. J^{mab}_{iej} contribution

$$R^{abcd}_{ijkl} \mathrel{-}= \sum_{\binom{ijkl}{i'j'/k'l'}} (\frac{1}{2} + P_{ac})[J^{mab}_{i'ej'} T^{ecd}_{k'ml'} + J^{mba}_{j'ei'} T^{ecd}_{k'ml'} + J^{mab}_{i'ej'} T^{edc}_{l'mk'} + J^{mba}_{j'ei'} T^{edc}_{l'mk'}] \ . \tag{S31}$$

K. K^{amn}_{ijk} contribution

$$R^{abcd}_{ijkl} \mathrel{+}= \sum_{\binom{ijkl}{i'j'/k'l'}} T^{abc}_{mnk'} K^{dmn}_{l'i'j'} + T^{abd}_{mnl'} K^{cmn}_{k'i'j'} \ . \tag{S32}$$

L. L^{abm}_{ijk} contribution

$$R^{abcd}_{ijkl} \mathrel{-}= \sum_{\binom{ijkl}{i'j'/k'l'}} P_{(i',j',k',l')}[L^{abm}_{i'j'k'} T^{cd}_{ml'} + L^{abm}_{i'j'l'} T^{dc}_{mk'}] \ . \tag{S33}$$

M. M^{abc}_{ejk} contribution

$$R^{abcd}_{ijkl} \mathrel{+}= \sum_{\binom{ijkl}{i'j'/k'l'}} P_{(i',j',k',l')}[T^{ea}_{j'i'} M^{bcd}_{ek'l'} + T^{eb}_{i'j'} M^{acd}_{ek'l'}] \ . \tag{S34}$$
